# The transformation mechanisms among cuboctahedra, Ino's decahedra and icosahedra structures of magic-size gold nanoclusters

**DOI:** 10.1039/d6na00012f

**Published:** 2026-05-19

**Authors:** Ehsan Rahmatizad Khajehpasha, Mohammad Ismaeil Safa, Nasrin Eyvazi, Marco Krummenacher, Stefan Goedecker

**Affiliations:** a Department of Physics, University of Basel Klingelbergstrasse 82 4056 Basel Switzerland stefan.goedecker@unibas.ch

## Abstract

Gold nanoclusters possess multiple competing structural motifs with small energy differences, enabling structural coexistence and interconversion. Using a high-accuracy machine learned potential trained on some 20 000 density functional theory reference data points, we investigate transformation pathways connecting both high-symmetry and distorted cuboctahedra, Ino's decahedra and icosahedra for Au_55_, Au_147_, Au_309_ and Au_561_ nanoclusters. Our saddle point searches reveal that high-symmetry transformations from cuboctahedra and Ino's decahedra to icosahedra proceed through a single barrier and represent soft-mode-driven jitterbug-type and slip-dislocation motions. In addition, we identify lower-barrier asymmetric transformation pathways that drive the system into disordered, Jahn–Teller–stabilized distorted icosahedra. Minima Hopping sampling further uncovers, in this context, many such low-symmetry minima. Some of the newly identified global minima for Au_309_ and Au_561_ have energies that are up to 2.8 eV lower than the previously reported global minima. Hence, both the shapes and the transformation pathways studied in previous investigations are not the physically relevant ones. In contrast to the previously studied pathways, our transformation pathways give reasonable transformation times that are in rough agreement with experiments.

## Abstract 2

Machine-learned interatomic potentials reveal that gold nanocluster transformations are not dominated by concerted symmetric pathways but by low-barrier localized transitions between distorted minima.

## Graphical abstract

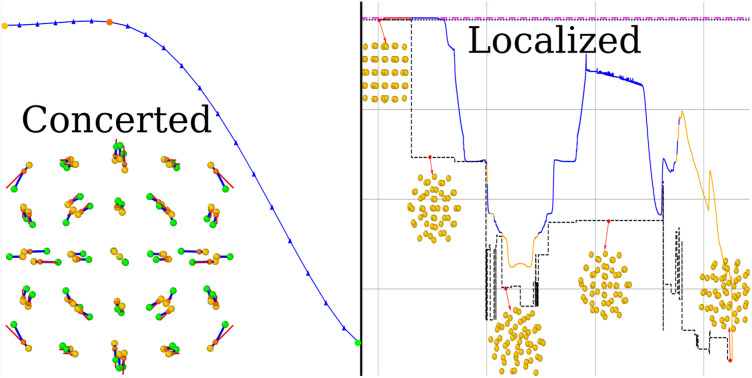

## 1 Introduction

Gold nanoclusters are of great interest due to their wide range of properties, enabling applications in many fields such as catalysis and biosensing.^1–3^ In addition to the geometric ground state, gold nanoclusters can also adopt a large number of low-energy metastable structures with properties that are possibly quite different from those of the ground state. These meta-stable structures can either be defective structures of the underlying ground-state structural motif or be based on completely different structures.^4^ Theoretically, for instance, it was found that a truncated octahedron Au_201_ is only 0.007 eV higher in energy than an icosahedron-like structure with five-fold symmetry.^5^ These small differences in energy between different structures are broadly consistent with experimental observations by the Palmer group.^6^

Larger nanoclusters are particularly stable if they consist of filled geometric shells. Such sizes are then called magic sizes. Icosahedra (*I*_h_) with 55, 147, 309, and 561 atoms consist of two, three, four, and five shells around the central atom and are thus magic sizes. For the same number of atoms, one can also obtain Ino's decahedra (*I-D*_5h_) and cuboctahedra (*O*_h_) with filled shells.

Foster *et al.*^6^ and Koga *et al.*^7^ concluded from their experiments that for Au_561_ nanoclusters, both the *I-D*_5h_ and face-centered cubic (FCC) structures, represented by the *O*_h_ motif, are energetically more favorable than *I*_h_, with *I-D*_5h_ being only 0.040 eV higher in energy than the FCC. Furthermore, they observed that at temperatures above 300 K, *O*_h_ and *I-D*_5h_ motifs are more prevalent than *I*_h_ on carbon supports. They also observed amazing dynamic inter-conversions between *I*_h_, *I-D*_5h_ and FCC-like structures occur on the time scale of seconds for nanoclusters with different numbers of atoms, such as Au_309_ (ref. 8) and Au_561_,^6^ highlighting the fluxional nature of these systems under experimental conditions.

In contrast, Density Functional Theory (DFT) calculations^9^ on isolated, ordered gold nanoclusters show that among the *I*_h_, *I-D*_5h_, and *O*_h_ structures, the *I*_h_ motif has the lowest energy, followed by *I-D*_5h_ and *O*_h_. This trend is attributed to the presence of high-energy (100) facets in *O*_h_ and *I-D*_5h_, making them energetically unfavorable, while rendering *I*_h_ the more stable structure.^10,11^ It is noteworthy that, although the *I*_h_ motif represents the ground state for magic-size Lennard-Jones (LJ) clusters and is the lowest-energy configuration among ordered structures, it does not correspond to the true ground state of gold nanoclusters. Instead, the Global Minima (GM) are structures closely related to the *I*_h_ geometry but incorporating slight distortions. This behavior was first identified for the Au_55_ nanocluster^12^ and subsequently observed for larger nanoclusters, including Au_147_, Au_309_, and Au_561_.^4^

The nature of the Au–C interaction explains the experimentally observed structural preferences. Gold on carbon supports typically exhibits weak binding energies of the order of ≈30–50 meV per atom, characteristic of physisorption with equilibrium separations around 3 Å.^13,14^ Under these conditions, both geometric constraints imposed by the carbon lattice and interfacial interactions play a decisive role in determining nanocluster morphology. Since the cluster structures are almost degenerate in energy, such a relatively weak binding to a substrate can still affect the energy ordering of the nanoclusters.

In particular, graphene and graphite substrates favour the formation of close-packed FCC or mixed FCC–HCP structures, while suppressing non-crystalline motifs such as *I*_h_. This behavior originates from the geometric matching between the hexagonal carbon lattice and the triangular arrangement of close-packed Au(111) layers, which enables flatter interfaces and better registry with the substrate. Consequently, supported nanoclusters tend to adopt layered geometries with predominantly (111) facets in contact with the carbon surface, while higher-energy facets such as (100) become unfavorable at the interface.^15^

Given the fact that *I*_h_, *I-D*_5h_, and *O*_h_ are fundamentally different, one might expect that the transformation from one structural motif to another proceeds by a nucleation process in which a large number of intermediate states are visited during the transformation. Schebarchov *et al.* calculated transformation pathways within Au_55_, Au_85_, and Au_147_ nanoclusters.^11^ Their paths cross several barriers while visiting about a dozen intermediate states with the lowest overall barriers of about 0.5 eV for Au_55_. They interpret this transformation pathway as partial melting followed by crystallization.

Plessow investigated the atomistic transformation pathways from *O*_h_ to *I*_h_ structures in copper and other metal nanoclusters and discovered that the transition can proceed through the so-called jitterbug transformation, requiring only a single barrier.^16^ He showed that for gold nanoclusters smaller than Au_309_, this barrier essentially vanishes, while for the Au_561_, it is approximately 2.8 eV at the DFT PBE + D3 level, based on a saddle point obtained with the Gupta potential.^17^

In this work, the meta-stable and ground state structures of gold nanoclusters with the magic sizes of 55, 147, 309, and 561 are re-examined. Then the transformations of *O*_h_, *I-D*_5h_, and *I*_h_ gold nanoclusters into each other, referred to as high-symmetry transformations from now on, are studied. We consider not only transformations leading to the *I*_h_ structure but also those resulting in various distorted forms—namely, distorted *I*_h_ (d-*I*_h_), distorted *I-D*_5h_ (d-*I-D*_5h_), and distorted *O*_h_ (d-*O*_h_). These will be referred to as asymmetric transformations throughout the remainder of this paper. Notably, similar distorted structures have previously been described as “amorphous” in ref. 4, although they retain identifiable symmetry motifs and are therefore characterized here as distorted.

## 2 Methods

**Fig. 1:**
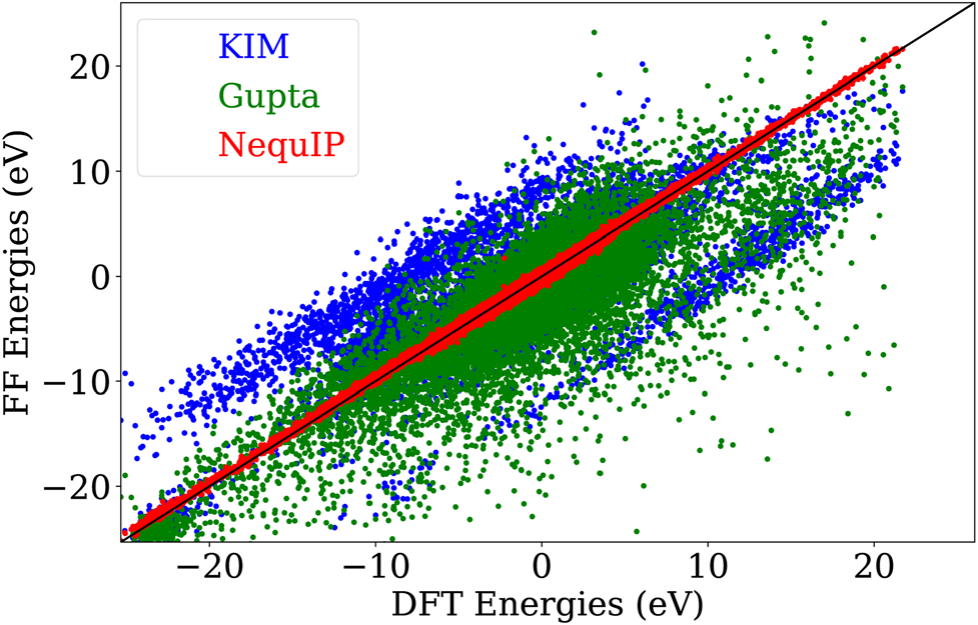
Correlation of the energies calculated by three different force fields for the structures in the training dataset of the MLIP.

DFT remains the method of choice for computing accurate energies and forces. Single-point calculations and geometry relaxations are now possible for nanoclusters containing hundreds of atoms. However, simulations requiring numerous energy and force evaluations—such as vibrational mode calculations, saddle point searches, or long-time Molecular Dynamics (MD) simulations remain computationally prohibitive for large nanoclusters on the DFT level. For this reason, such theoretical investigations of large nanoclusters were done nearly exclusively by approximate interatomic potentials such as the Gupta potential or MEAM^18,19^ methods. The price for the gain in speed is reduced accuracy. Fig. 1 illustrates the poor agreement between the energies obtained from DFT on the one hand and either the Gupta or MEAM potential on the other hand over a diverse dataset of 19 383 gold nanoclusters.

The advances in the development of machine-learned interatomic potentials (MLIPs) have changed the situation dramatically. MLIPs can nowadays give DFT accuracy at a small fraction of the DFT cost, enabling complex simulations of large systems. We therefore use such an MLIP, namely NequIP,^20–22^ to investigate large gold nanoclusters. The MLIP was trained on a dataset of 19 383 nanoclusters, each containing between 10 and 90 atoms. The DFT reference energies were computed with the Projector-Augmented Wave (PAW) method^23,24^ using 11 valence electrons as implemented in the Vienna *Ab initio* Simulation Package (VASP)^25,26^ together with the Perdew–Burke–Ernzerhof (PBE) exchange-correlation functional.^27^ To remove interactions between periodic images, a vacuum of 12 Å was introduced in all directions around the nanoclusters.

As shown in Fig. 1, the resulting MLIP exhibited a root mean square error of 7.9 meV per atom for energies and 75.4 meV Å^−1^ for forces, demonstrating excellent accuracy for the subsequent tasks. To further assess the transferability of the MLIP, we evaluated its performance on larger nanoclusters—Au_55_, Au_147_, Au_309_, and Au_561_—that were not included in the training set. The model yielded a root mean square error of 5.2 meV per atom for energies (see Fig. S3) and 37.9 meV Å^−1^ for forces. MLIP forces and energies were used for MD, saddle point searches with both the Nudged Elastic Band (NEB)^28^ and COMPASS,^29^ Minima Hopping method (MH) runs^30–34^ and vibrational mode analysis within the Atomic Simulation Environment (ASE).^35^ The training set was generated iteratively using MD and MH simulations combined with active learning, with the MLIP training set expanded after each iteration. The resulting dataset spans an energy range of up to 1.12 eV per atom. The energy and size distributions of the training structures are shown in the SI, Fig. S1 and S2, respectively.

MH explores the PES by going over low barriers and visiting low-energy local minima in the pursuit of finding the GM. By steering the MH search towards a target structure, a collection of minima can be obtained to connect the initial structure to the target structure with a physically realizable pathway.^36^ The steering is obtained by performing MH on a biased PES (biased MH) *Ẽ*(**R**) = *E*(**R**) + *ωD*(**R**, **R**^tar^) where *ω* is the bias weight and *D*(**R**, **R**^tar^) = |*F*(**R**) − *F*(**R**^tar^)|^2^ is a distance function that is zero if the current structure **R** is equal to the target structure **R**^tar^ and positive for all other structures.

The fingerprint vector **F**(**R**) contains the eigenvalues of the Laplace matrix *L*. Position-dependent matrix elements are given by:1
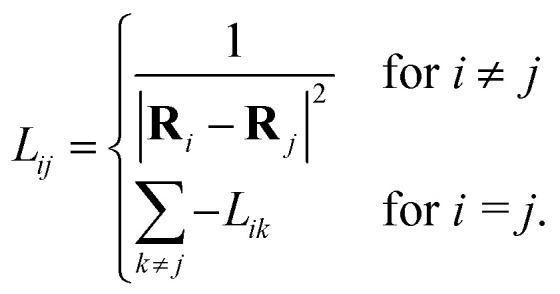


Since the length of a fingerprint vector **F** is *N* for a nanocluster containing *N* atoms, it can actually not uniquely specify the 3*N* degrees of freedom of all the atoms. However, for a biasing scheme, this is not problematic.

To determine the transformation pathway between different atomic configurations of gold nanoclusters, NEB and COMPASS calculations were conducted. The mapping of atomic indices of the initial and final structures in COMPASS calculations for asymmetric transformations was determined by a simple Root Mean Square Distance alignment (RMSD).^37^ The parameters of COMPASS are provided in table SV. For the high-symmetry transformations, a total of 21 images was picked, including the initial and final configurations for each NEB pathway from biased MH. The NEB calculations converged to a force tolerance of five meV Å^−1^, using the Fast Inertial Relaxation Engine (FIRE),^38^ ensuring high precision in the computed transition states and reaction pathways. To calculate the vibrational frequencies and modes, a five-point finite difference method with a finite displacement of 0.01 Å was employed to compute the Hessian matrix.

The low energy barriers determine the dynamics of a system at ambient temperatures. Empirical findings suggest that MD trajectories starting from a minimum along a soft vibrational mode are more likely to cross low-energy barriers compared to those starting in other directions.^39^ The softest mode on the PES corresponds to the lowest non-zero vibrational frequency and reveals the most transformation-prone direction in configuration space. By aligning the initial velocities along this mode and scaling their magnitude to correspond to a specific temperature, one can control both the energy input and its directional bias, effectively triggering rapid structural transformations. In a related context, Wang *et al.*^40^ studied titanium oxide nanoparticles under negative pressure and found also that soft vibrational modes can trigger coherent structural phase transformations.

According to classical transition state theory, the reaction rate, *k*, for crossing a barrier is given by:2
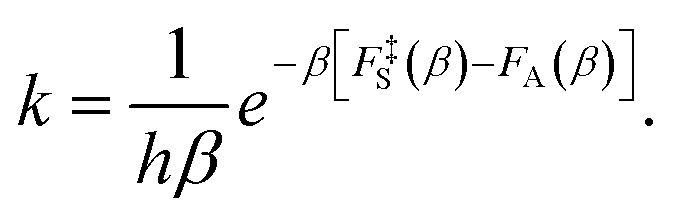

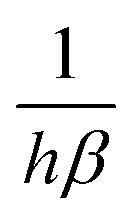
 is the attempt frequency that can be considered as the rate with which the system tries to overcome a barrier at temperature *T* with numerical value of 6.25 × 10^12^ (Hz) at *T* = 300 K. *F*^‡^_S_(*β*) is the free energy of the saddle point and *F*_A_(*β*) is the free energy of the local minimum. The ‡ symbol indicates the usage of positive modes only when calculating the free energy. The six zero modes resulting from rotational and translational symmetries do not contribute. In our calculations, we approximate the free energy by the energy.

## 3 Results and discussion

### 3.1 Equilibrium structures

To thoroughly sample the PES of Au_55_, Au_147_, Au_309_ and Au_561_, we used MH combined with MLIP without any biasing. From the ensemble of the visited minima, we selected the twelve lowest-energy candidates. The selected candidates were further relaxed up to one meV Å^−1^ using VASP with ISPIN = 2 to enable spin-polarized calculation, ISMEAR = 0 for Gaussian smearing with width SIGMA = 0.0005 eV. The small SIGMA value is chosen because we are interested in the zero temperature limit where Jahn–Teller distortions occur in our nanoclusters.^41^

**Fig. 2:**
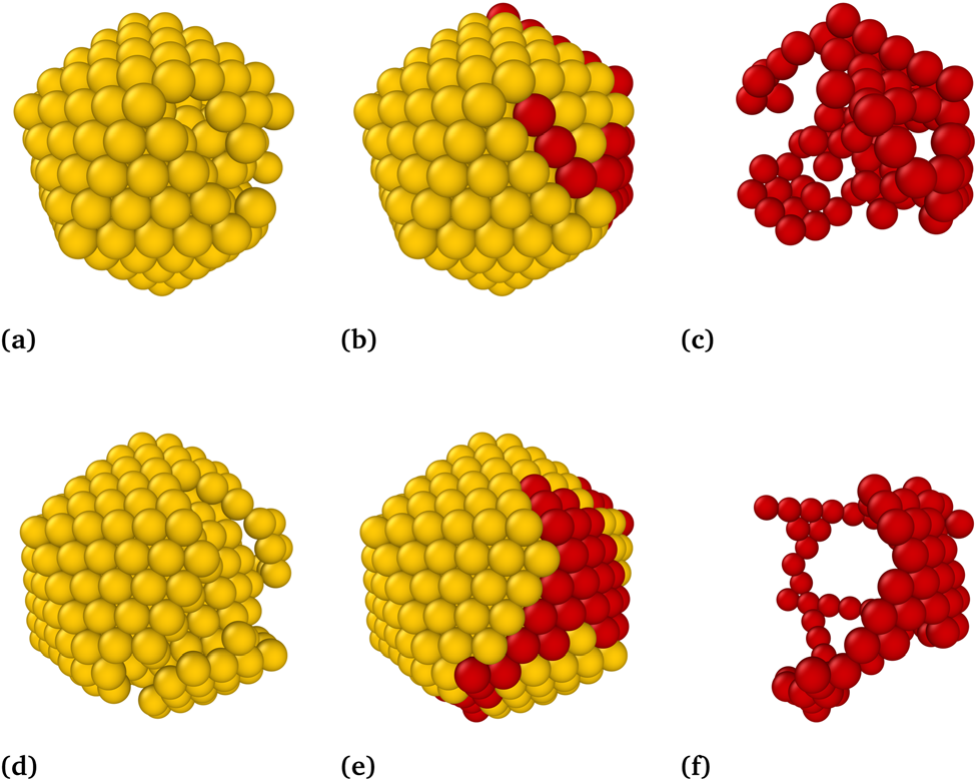
New GM structures for Au_309_ (top row) and Au_561_ (bottom row). (a and d) Atoms whose positions coincide with those of an *I*_h_ are shown in gold. (b and e) Complete nanocluster structures, where ordered (*I*_h_-matching) atoms are shown in golden color and distorted atoms are shown in red. (c and f) Isolated views of the distorted (red) atoms extracted from the corresponding structures.

Table 1 shows that the newly identified global minima of Au_309_ (Fig. 2b) and Au_561_ (Fig. 2e) are 0.260 and 2.896 eV lower in energy than the previously reported structures,^4,42^ even though their overall morphologies remain largely unchanged. The structures have *I*_h_ character, displaying well-defined triangular facets on one side and a somewhat distorted character on the other side. We found some 200 structures of this d-*I*_h_ type, which suggests that the *I*_h_ funnel dominates the low-energy landscape. Alternative motifs remain, however, competitive in energy (Table 1). The coordinates of the 10 lowest-energy distorted structures are provided in the SI.

**Table 1:** Energy differences relative to the geometry optimized *I*_h_ structure (in eV). Our lowest energy structures are contrasted with those from ref. 4

Rank	Au_55_	Au_147_	Au_309_	Au_561_
d-*I*_h_ 1	−2.387	−4.382	−8.625	−13.455
d-*I*_h_ 2	−2.385	−4.304	−8.574	−13.395
d-*I*_h_ 3	−2.353	−4.243	−8.412	−13.334
d-*I*_h_ 4	−2.318	−4.189	−8.396	−13.306
d-*I*_h_ 5	−2.311	−4.187	−8.224	−13.305
Ref. 4	a	a	−8.366	−10.559
*O*_h_	1.085	3.110	4.802	3.536
*I-D*_5h_	0.434	2.518	1.173	1.024

a Ref. 4 does not present the structures of Au_55_ and Au_147_.

**Fig. 3:**
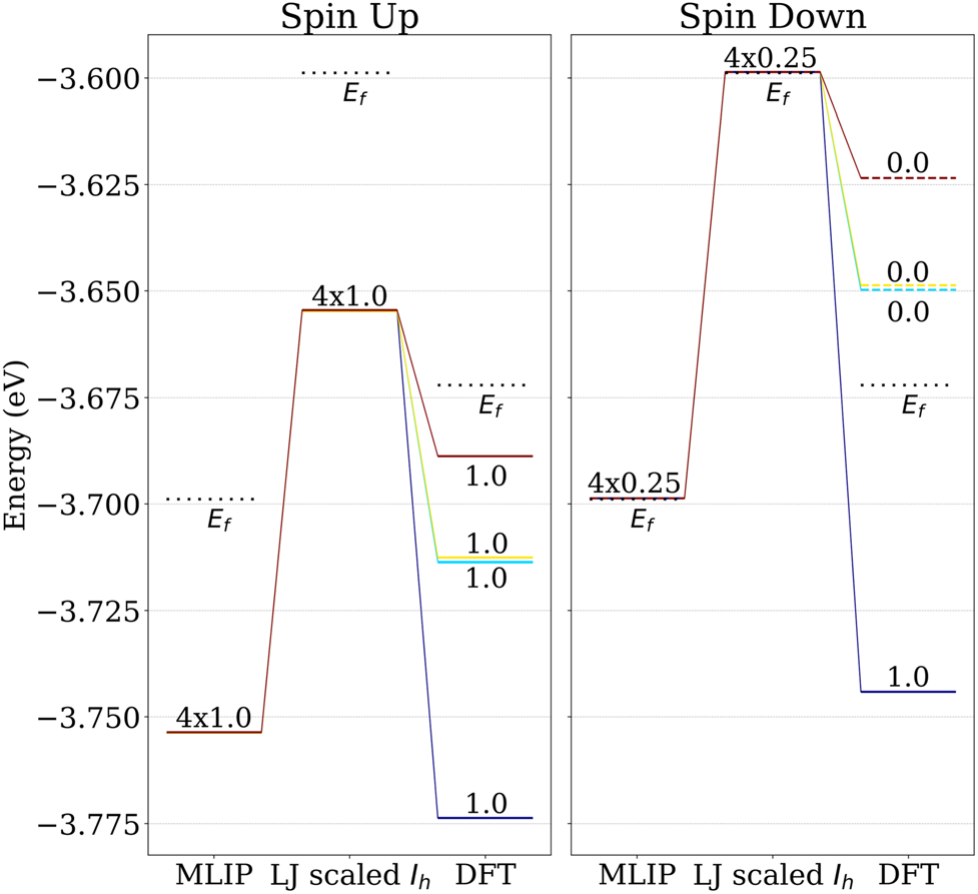
Energy levels near the Fermi level calculated for three different structures of Au_55_-*I*_h_ obtained with three approaches: (i) scaling LJ_55_-*I*_h_ cluster on the MLIP PES with *a* factor (scaled LJ), (ii) relaxing the scaled LJ structure on the MLIP PES (MLIP-relaxed), and (iii) further relaxing the MLIP-relaxed structure on the DFT PES (VASP-relaxed). The scaling factor in approach (i) was the one that gave the lowest MLIP energy. Occupied and unoccupied levels are shown as solid and dashed lines, respectively, while the Fermi level is indicated by a black dotted line.

Fig. 3 shows that while a well-trained MLIP can capture the overall features of the energetic landscape of gold nanoclusters, it lacks quantum-mechanical principles to account for subtle electronic effects such as a lowering of the occupied levels or Jahn–Teller distortions, which are known to play a decisive role in stabilizing lower-energy isomers *via* symmetry breaking.^43^ Such effects are, for instance, observed when the MLIP-relaxed *I*_h_ was tightly DFT post-relaxed while its electronic structure was calculated using VASP with *I*SPIN = 2, ISMEAR = 0 and SIGMA = 0.0005.

**Fig. 4:**
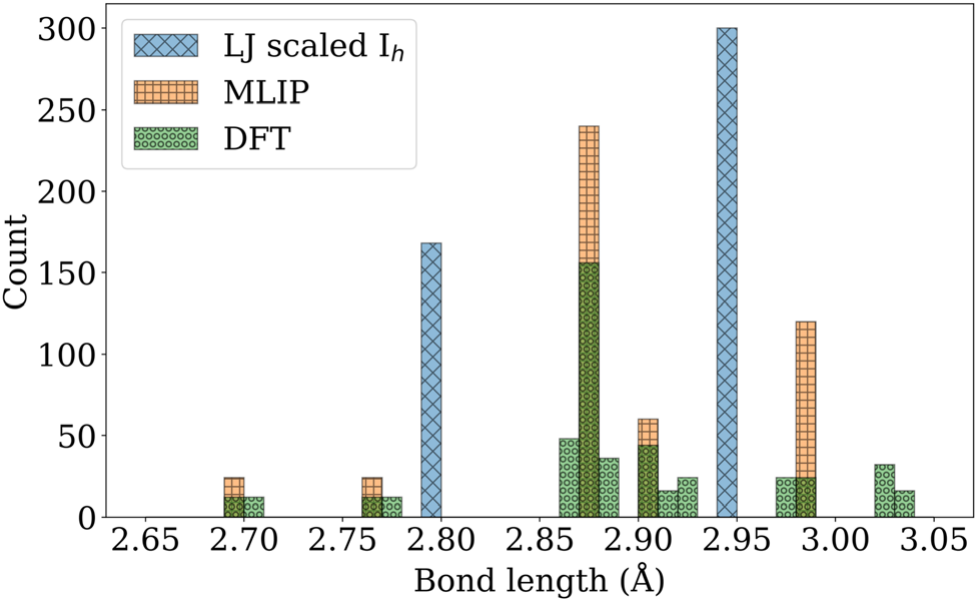
Histogram comparing the bond length distributions of the Au_55_-*I*_h_ nanocluster as obtained from scaled LJ, MLIP-relaxed, and VASP-relaxed structures.

While the MLIP-relaxed *I*_h_ structure preserves degenerate electronic levels, these are lifted upon further relaxation of forces up to one meV Å^−1^ in spin-polarized DFT, indicating a Jahn–Teller distortion.^43^ This lifting of degeneracy enables complete filling of the resulting split levels, as shown in Fig. 3 for Au_55_-*I*_h_. The accompanying changes in the Au–Au bond lengths and the resulting broadening of their distribution are shown in Fig. 4. The total energy of the MLIP-relaxed Au_55_-*I*_h_ nanocluster decreased by 0.046 eV during this Jahn–Teller distortion, and the largest atomic displacement observed during this step was 0.053 Å. The breaking of the full *I*_h_ symmetry is therefore hard to detect by eye, and we will continue to refer to the Jahn–Teller distorted structure as the *I*_h_ for the rest of the paper.

The same effect exists for all magic *I*_h_ sizes, although the degree of gap opening decreases with size as the density of states near the Fermi level increases. So, the rule established for smaller nanoclusters,^44^ namely that nanoclusters adopt a structure that allows them to fill degenerate levels completely, is also valid for larger nanoclusters, even though their overall shape is dictated by geometric effects.

**Fig. 5:**
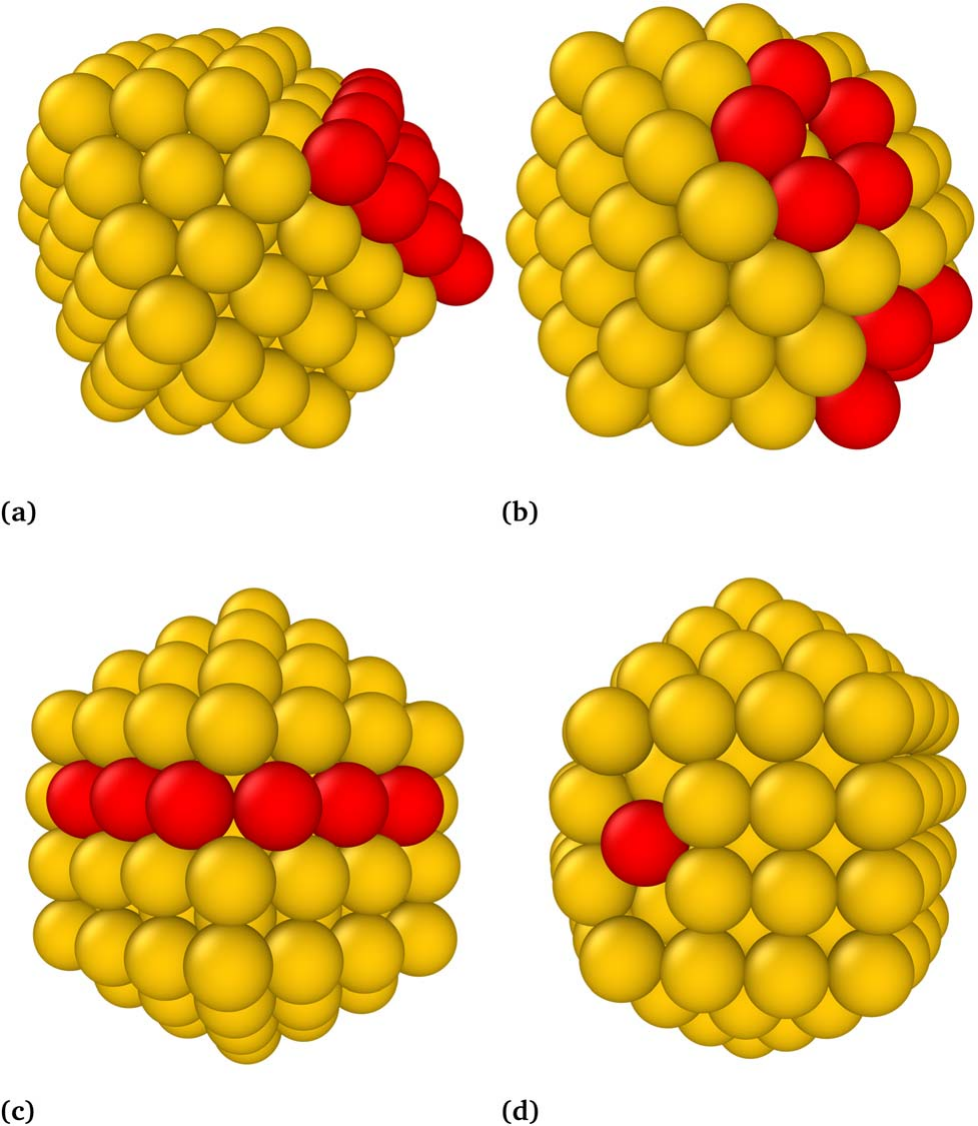
Examples of two distorted *O*_h_-Au_147_ (a and b) and two *I-D*_5h_-Au_147_ (c and d). Red spheres indicate atoms that deviate from their ideal positions in the corresponding high-symmetry nanocluster.

In analogy to the *I*_h_, where distorted *I*_h_ nanoclusters were lower in energy than the *I*_h_, there also exist d-*I-D*_5h_ and d-*O*_h_ that are lower in energy than their high symmetry structures. Four such structures are shown in Fig. 5. The number of distorted *I-D*_5h_ and *O*_h_ that are lower in energy than their high-symmetry counterparts is smaller than in the case of the *I*_h_.

### 3.2 Minimum energy transformation pathways

Performing biased MH runs where the target structure was *I*_h_, revealed immediately the so-called Jitterbug transformation for elastic bodies connecting *O*_h_ → *I*_h_ and a cooperative slip-dislocation mechanism reported for an elastic body by Koga *et al.* for the *I-D*_5h_ → *I*_h_ transformation.^7^ In this way, we could show that these transformations, which were only defined for shapes, can also be represented by simple and short atomic trajectories.

**Fig. 6:**
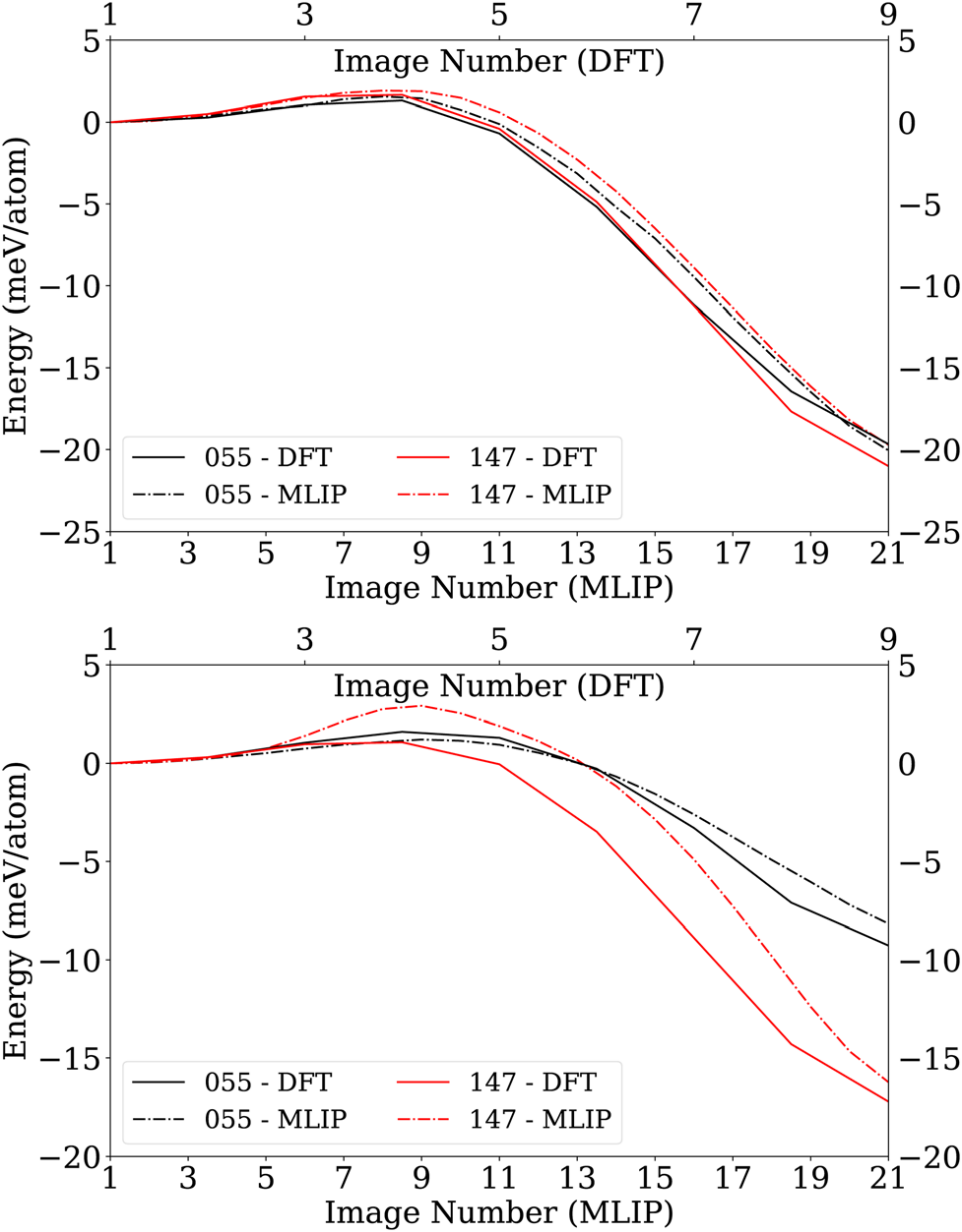
Comparison of energy profiles along the transformation pathways for Au_55_ and Au_147_ nanoclusters obtained from NEB calculations for *O*_h_ → *I*_h_ and *I-D*_5h_ → *I*_h_ transformations (top and bottom, respectively). The energy of structures along the transformation pathway remains nearly the same, independently of whether single-point DFT energies of MLIP-relaxed images are calculated (dashed line) or the NEB is fully relaxed directly at the DFT level (solid line).

To assess the accuracy of the MLIP for structural transformations, NEB calculations were performed for Au_55_ and Au_147_ on the DFT PES with an initial guess consisting of nine images selected from biased MH. For comparison, NEB calculations were also performed with 21 images on the MLIP PES. The NEB was relaxed up to ten meV per atom on DFT PES and five meV per atom on MLIP PES. Solid lines in Fig. 6 show the energy of NEB images on the DFT PES with no climbing image, *i.e.*, intermediate image that feels no forces from the NEB springs. Dash-dotted lines represent the MLIP energy profiles for Au_55_ and Au_147_ nanoclusters along high-symmetry transformations, including a climbing image. The agreement between the two methods confirms that the MLIP reproduces DFT-level energetics throughout these transformations within the MLIP error range of 7.9 meV per atom. Having established the accuracy of MLIP, we subsequently employed the MLIP to perform NEB calculations using 21 equally spaced structures for high-symmetry transformations of Au_309_ and Au_561_ nanoclusters.

**Fig. 7:**
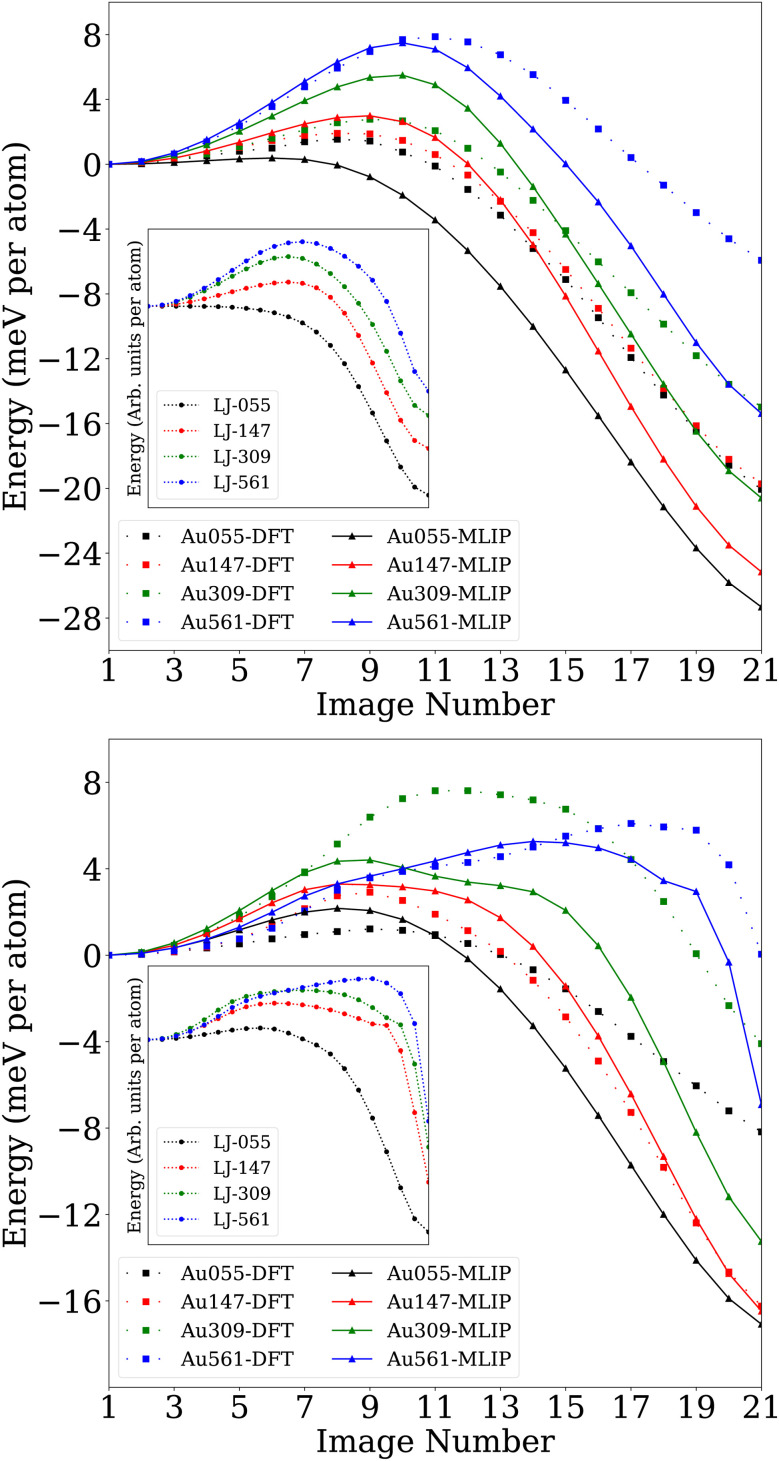
The NEB pathway calculated by MLIP, dash-dotted line, for *O*_h_ → *I*_h_ and *I-D*_5h_ → *I*_h_ transformation, top and bottom, respectively. The single-point energies of NEB individual images calculated on DFT PES are represented by a dotted line. In the insets, the relevant NEB energy pathway for geometrically scaled and relaxed structures on the LJ potential is presented.

Fig. 7 shows that NEB paths indeed connect the two structures while crossing only one barrier. However, initial vibrational calculations using MLIP indicated that the identified saddle points, *i.e.*, the climbing images of the NEB, were not first-order. In this case, it should be possible to find lower-energy first-order saddle points. Such lower saddle points on the high-symmetry transformation pathway were, however, not found. To eliminate the possibility that the second-order character of the saddle points is an artifact of numerical noise, which is always present in MLIP or DFT calculations, and to isolate purely geometric effects, we additionally performed NEB simulations using a simple LJ potential, which is noise-free.

The tests were carried out on scaled geometries of the gold *I-D*_5h_, *O*_h_, and *I*_h_ structures. The LJ transformation pathways were virtually (up to a scaling factor) identical to those of the gold nanoclusters, showing that the jitterbug and slip-dislocation transformations are purely geometrical and largely independent of the interaction potential. A similar behavior is observed for ionized Au_55_: the geometric pathway of the high-symmetry transformation remains essentially unchanged, while the barrier heights remain small (see Section S3). In the limit of large nanoclusters, the transformation pathways and barrier heights are expected to become even less sensitive to the charging of the nanocluster. The additional low-curvature directions with multiple negative eigenvalues, observed on the MLIP PES, were not present when the Hessian was calculated analytically for the corresponding LJ saddle points. This strongly suggests that the extra negative modes in the MLIP results stem from numerical noise in the MLIP PES.

**Fig. 8:**
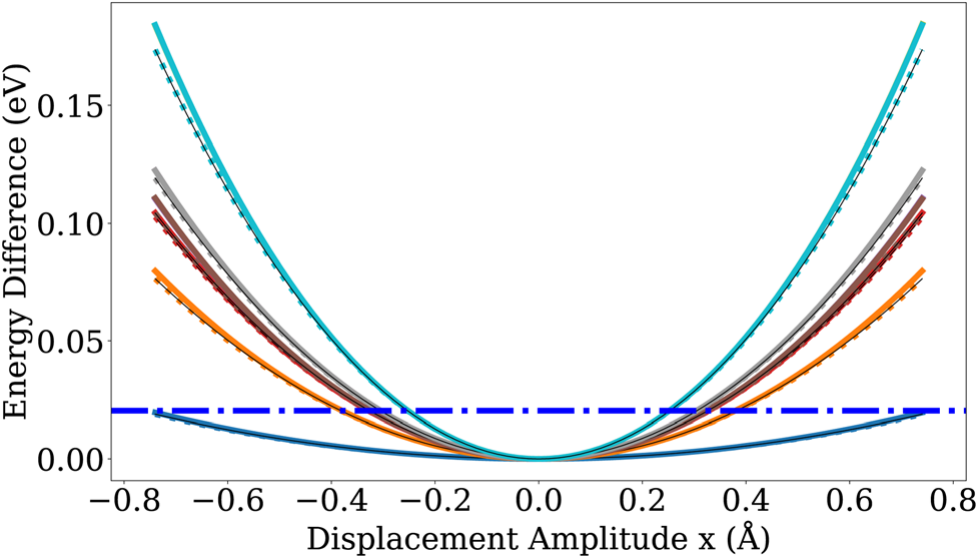
Energy for displacements along vibrational modes of Au_55_-*I-D*_5h_. The solid lines show the energy profiles when displacing atoms along the ten non-zero lowest-frequency (softest) modes. For comparison, the dotted lines of the same color indicate the energy of the corresponding harmonic potential for each mode. The dash-dotted blue line represents the energetic distance of the highest-frequency vibrational modes, *ħω*_max_. The fact that this line is at room temperature below *k*_B_*T* ≈ 0.025 eV shows that all vibrational modes are thermally accessible at room temperature.

**Fig. 9:**
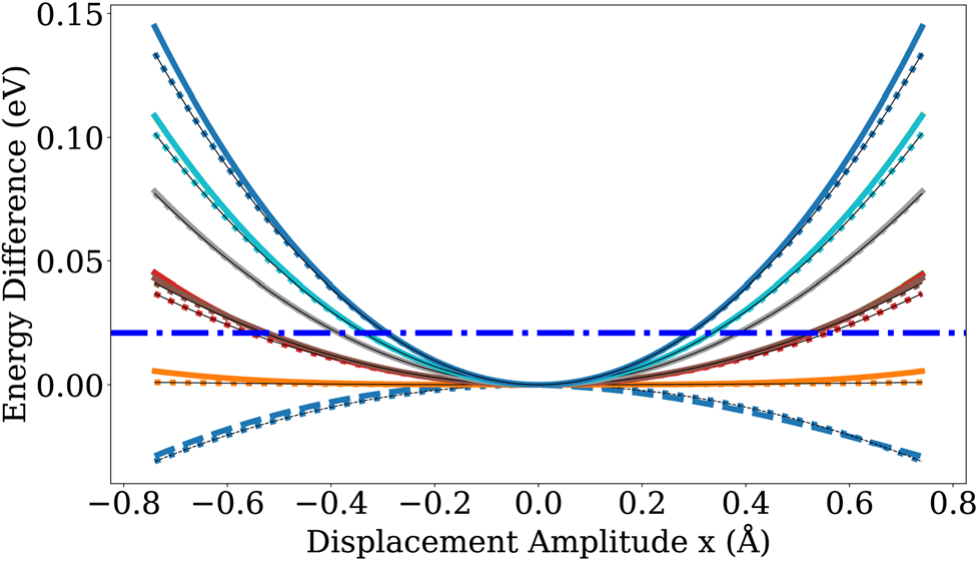
Energy for displacements along vibrational modes of the saddle point of the Au_55_-*I-D*_5h_ → Au_55_-*I*_h_ transformation. The conventions are the same as in Fig. 8. The additional dashed line denotes the imaginary mode at the saddle point, arising from a negative curvature of the PES along this mode.

It is also worth noting in this context that displacements along the lowest positive curvature direction of the LJ saddle points revealed a wide and nearly flat valley in the saddle point region with a shape similar to the one of the gold PES depicted in Fig. 8 and 9. This flatness can introduce significant numerical noise when estimating vibrational frequencies *via* finite differences on the MLIP PES, complicating the accurate characterization of saddle points. The energy profile along this direction, computed for the saddle points of the LJ and gold nanoclusters, is provided in Fig. S6.

As can be seen from Fig. 7, the barrier height of small nanoclusters is almost negligible, but it increases as the nanoclusters grow larger, reaching approximately 4.2 eV for Au_561_-*O*_h_. The relaxed pathways indicate that the transformations from *O*_h_ and *I-D*_5h_ to *I*_h_ are strongly exothermic, with the degree of exothermicity decreasing as the nanocluster size increases. In our calculations, the energy pathway for the *O*_h_ → *I*_h_ transformation (Fig. 7) remains qualitatively consistent across all investigated nanocluster sizes. The energy profiles obtained here are similar to the transformation pathway reported by Plessow^16^ for copper nanoclusters, although the energy barriers we observe are higher. Specifically, our calculated activation energies for the *O*_h_ → *I*_h_ transformation are higher than those reported by Plessow by 0.440, 1.698, and 1.402 eV for Au_147_, Au_309_, and Au_561_, respectively. This difference can most likely be attributed to the improved accuracy of the MLIP employed in this work compared to the Gupta potential used by Plessow.^16^

The NEB pathway of the LJ system, shown in the insets of Fig. 7, also follows the same trend as the gold MLIP results despite the absence of any bond-bending terms in the pairwise LJ potential. This indicates that the transformations are predominantly geometry-driven and largely independent of the material.

**Fig. 10:**
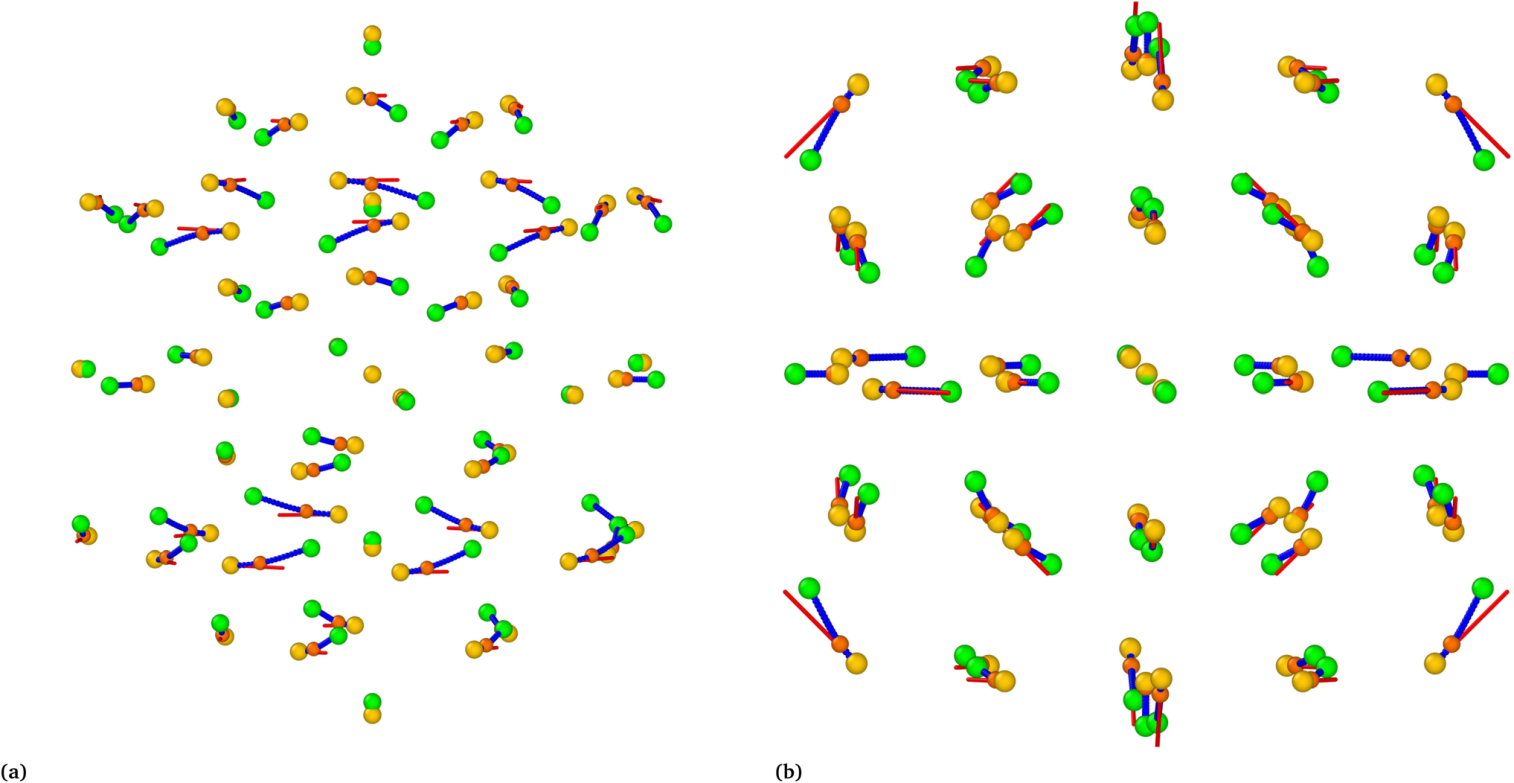
(a) Depicts the high-symmetry Au_55_-*I-D*_5h_ → Au_55_-*I*_h_ transformation pathway (side view), while (b) illustrates the high-symmetry Au_55_-*O*_h_ → Au_55_-*I*_h_ pathway (viewed along (100)). Golden spheres represent the initial structures (*I-D*_5h_ or *O*_h_), green spheres indicate the final structure (*I*_h_), red lines show the direction of the softest mode, blue lines trace the NEB transformation pathway, and orange highlights the saddle-point configuration. The *xyz*-coordinates of these pathways are provided in the SI.

Fig. 10 provides a visual representation of the minimum energy pathway for the high-symmetry transformations of Au_55_ alongside the lowest vibrational modes. The results for other sizes, shown in Fig. S8, follow almost the same trend as Au_55_ nanoclusters. The xyz-coordinates of atoms for the transformation pathways are provided in the SI. In Fig. 10, one can clearly see that the high-symmetry transformation is not a nucleation process. Instead, it is a concerted, smooth, simultaneous movement of all atoms. At the starting local minimum, the tangent to the minimum energy pathway is well aligned with the lowest-frequency vibrational mode, indicating that the system predominantly evolves along the softest mode direction during a high-symmetry transformation.

To determine the lowest barrier that a high-symmetry nanocluster must overcome to transform into the GM, we employed the COMPASS method.^29^ The complex transformation pathway identified by COMPASS clarified why the NEB method fails when applied to the asymmetric transformation. Interestingly, barriers leading into these low-energy distorted structures were lower than those leading to the *I*_h_. As a result, it is much more likely that the system will transform into a d-*I*_h_ than into the *I*_h_. Experimentally, one can presumably not distinguish the two forms of *I*_h_.

**Fig. 11:**
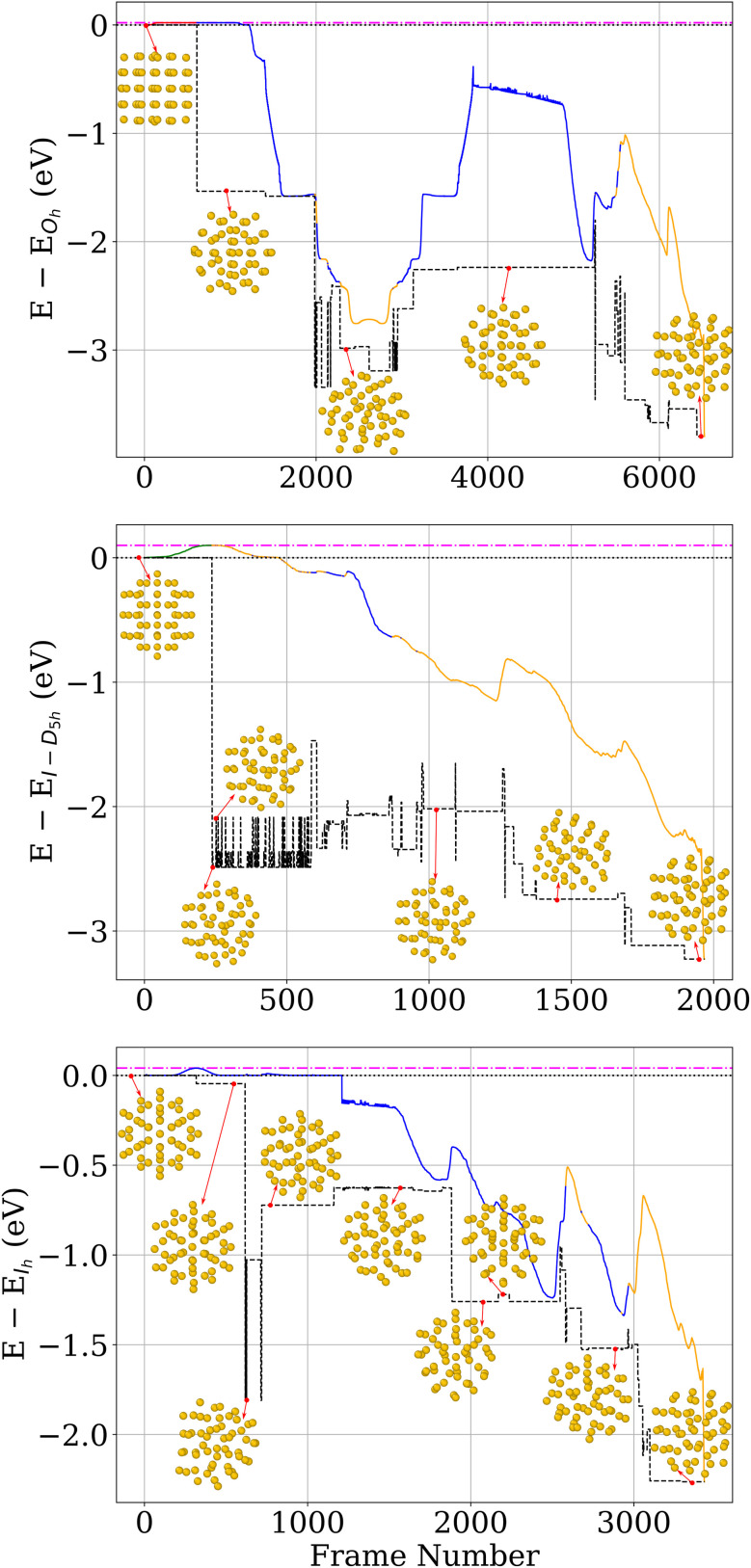
Energy profiles along the COMPASS transformation pathways are shown as colored solid lines. The top, middle, and bottom panels correspond to pathways starting from *O*_h_, *I-D*_5h_, and *I*_h_, respectively. Each curve displays the energy difference relative to the starting structure of its panel. The dotted black line denotes zero energy. The dash-dotted magenta line marks the energy of the highest-energy structure along the pathway, indicating the upper bound of the barrier. The dashed black line shows the catchment basin visited during the transformation, obtained by relaxing the COMPASS pathway structures on the MLIP PES. Colors on the solid line indicate the structural motif: red for *O*_h_, green for *I-D*_5h_, blue for *I*_h_, and orange for fully distorted nanoclusters.

**Fig. 12:**
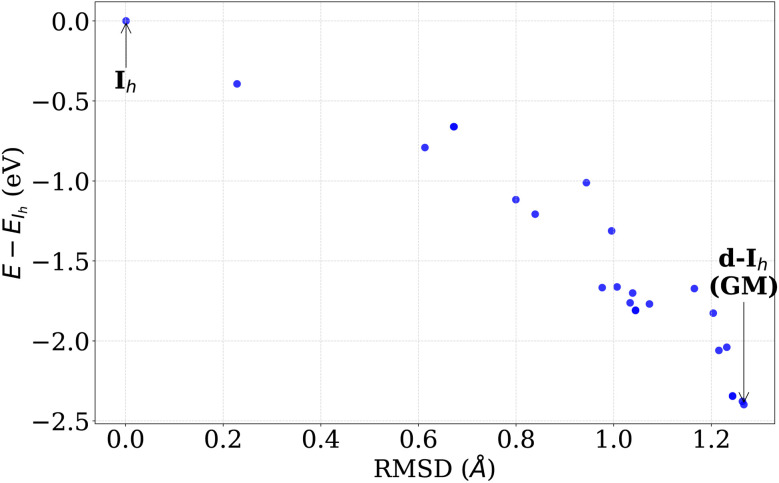
Comparison of energy difference relative to the *I*_h_ and RMSD from the *I*_h_ structure for Au_55_ nanoclusters. Points represent minima sampled within the *I*_h_ funnel. The GM (d-*I*_h_) corresponds to a d-*I*_h_ structure with lower energy than the *I*_h_.

Pathways that lead into the GM are complicated, as shown in Fig. 11. Multiple barriers of various heights have to be crossed to reach the GM. To quantify the symmetry breaking associated with the stabilization of these d-*I*_h_ structures relative to the *I*_h_ geometry, we analyze the energy and the structural deviation from the *I*_h_, measured by RMSD from the *I*_h_ structure. As shown in Fig. 12, the energy decreases with increasing RMSD, indicating that lower-energy configurations are obtained *via* distortions of the *I*_h_ structure. The magnitude of these distortions remains limited, such that the resulting configurations preserve the overall *I*_h_-like morphology while lacking exact symmetry.

The asymmetric transformations do not proceed along a single dominant vibrational mode. When expressing the initial atomic displacement by a linear combination of the vibrational modes, soft modes contribute most significantly to this motion, whereas higher-frequency (hard) modes play a minor role. To quantify this relationship, we compute the angle between the vector connecting the initial structure to the asymmetric transformation saddle point and each vibrational eigenmode. The resulting angular distribution, shown in Fig. S7 illustrates how individual vibrational modes participate in shaping the reaction direction at the beginning of asymmetric transformations.

In Table 2, the movement amplitudes of atoms during various transformations are presented. In the inner shells, the atoms move only by a small fraction of a bond length, while in the outer shells, the movement is larger, reaching 1.15 times the gold bond length in nanoclusters, *i.e.*, 2.94 Å.

**Table 2:** Minimum and maximum atomic displacements (in Å) observed during various transformations, excluding the innermost atom

Transformation		Au_55_	Au_147_	Au_309_	Au_561_
*O*_h_ → *I*_h_	Min	0.4	0.4	0.4	0.4
	Max	1.3	1.9	2.6	3.2
*O*_h_ → GM	Min	0.1	0.1	0.1	0.1
	Max	2.6	2.4	2.7	3.2
*I-D*_5h_ → *I*_h_	Min	0.1	0.2	0.2	0.2
	Max	1.5	2.3	3.1	3.9
*I-D*_5h_ → GM	Min	0.1	0.1	0.1	0.1
	Max	2.3	2.9	2.9	3.0
*I*_h_ → GM	Min	0.1	0.1	0.1	0.1
	Max	2.8	3.1	3.3	3.4

Portales *et al.* attributed a ∼200 GHz shift in low-frequency Raman scattering to quadrupolar (*E*_g_ and *T*_2g_) vibrational modes in 4.3–5.3 nm gold nanocrystals.^45^ Although these nanocrystals contain far more atoms than our nanoclusters, their reported *T*_2g_ mode aligns with the softest vibrational modes of the *O*_h_ structures listed in Table 3. Notably, the *T*_2g_ mode described by Portales *et al.* corresponds closely to the jitterbug motion associated with the *O*_h_ → *I*_h_ transformation. Still, it differs significantly from the slip-dislocation twisting involved in the *I-D*_5h_ → *I*_h_ transformation. The red lines, direction of *O*_h_ softest mode, in Fig. 10b align with the arrows showing the *T*_2g_ mode in the Portales paper.

**Table 3:** Energy (in meV) and frequencies (in GHz) of the softest modes for *O*_h_, *I-D*_5h_ and *I*_h_

	*O*_h_		*I-D*_5h_		*I*_h_	
	*E*	*f*	*E*	*f*	*E*	*f*
Au_55_	0.8	200.86	1.2	290.80	2.5	602.58
Au_147_	1.2	287.80	1.1	263.81	2.1	500.65
Au_309_	1.1	278.80	0.9	227.84	1.7	398.72
Au_561_	1.1	254.82	0.8	200.86	1.3	323.77

### 3.3 Kinetics of transformation

The barriers for all kinds of transformations in the small Au_55_ and Au_147_ nanoclusters are quite low, as shown in Table 4. Classical transition state theory gives transformation times of the order of picoseconds (ps) for these barriers, consistent with predictions by Schebarchov *et al.*^11^ Experimental transformation rates are, however, of the order of seconds. This discrepancy is likely due to the interaction between the nanoclusters and the substrate, as Foster *et al.* noted in their SI.^6^ Presumably, the interaction of the nanocluster with the substrate makes rearrangements harder. For the larger nanoclusters, the transition times explode, whereas the experimental time scales remain nearly constant.^46^ It is hard to believe that the interaction with a substrate can speed up transformations in a nanocluster that is unwilling to transform.

**Table 4:** Barrier heights (in eV) for both high-symmetry and asymmetric transformations. The 
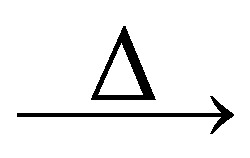
 shows the barrier heights extracted from Van't Hoff plots based on the standard MD data (see Fig. 13). Values in square brackets represent the resulting lifetime of initial nanoclusters in ps using transition-state theory at 300 K for these barriers

Transformation	Au_55_	Au_147_	Au_309_	Au_561_
*O*_h_ → *I*_h_	2.09 × 10^−2^	4.40 × 10^−1^	1.70 × 10^0^	4.20 × 10^0^
	3.6 × 10^−1^	3.9 × 10^6^	5.8 × 10^27^	5.8 × 10^69^
*O*_h_ → d-*O*_h_	2.09 × 10^−2^^a^	2.28 × 10^−1^	2.91 × 10^−1^	3.62 × 10^−1^
	3.6 × 10^−1^	1.1 × 10^3^	1.2 × 10^4^	1.9 × 10^5^
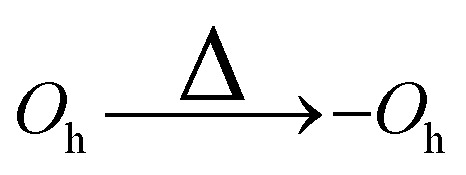	4.55 × 10^−2^	3.33 × 10^−1^	2.44 × 10^−1^	1.74 × 10^−1^
	9.3 × 10^−1^	6.3 × 10^4^	2.0 × 10^3^	1.3 × 10^2^
*I-D*_5h_ → *I*_h_	1.19 × 10^−1^	4.84 × 10^−1^	1.36 × 10^0^	2.95 × 10^0^
	1.6 × 10^1^	2.1 × 10^7^	1.1 × 10^22^	5.8 × 10^48^
*I-D*_5h_ → d-*D*_5h_	9.96 × 10^−2^	1.60 × 10^−1^	1.92 × 10^−1^	2.15 × 10^−1^
	7.6 × 10^0^	7.8 × 10^1^	2.7 × 10^2^	6.6 × 10^2^
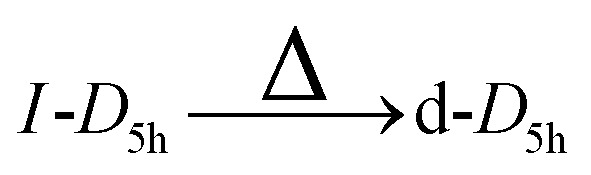	1.74 × 10^−1^	2.29 × 10^−1^	2.29 × 10^−1^	2.13 × 10^−1^
	1.4 × 10^2^	1.1 × 10^3^	1.1 × 10^3^	6.2 × 10^2^
*I*_h_ → *O*_h_	1.55 × 10^0^	4.23 × 10^0^	8.26 × 10^0^	13.22 × 10^0^
	2.2 × 10^25^	1.8 × 10^70^	9.2 × 10^137^	2.0 × 10^221^
*I*_h_ → *I-D*_5h_	1.08 × 10^0^	2.99 × 10^0^	5.68 × 10^0^	8.82 × 10^0^
	2.4 × 10^17^	3.8 × 10^49^	5.3 × 10^94^	3.3 × 10^147^
*I*_h_ → d-*I*_h_	4.13 × 10^−2^	1.75 × 10^−1^	2.06 × 10^−1^	5.44 × 10^−1^
	7.9 × 10^−1^	1.4 × 10^2^	4.7 × 10^2^	2.2 × 10^8^
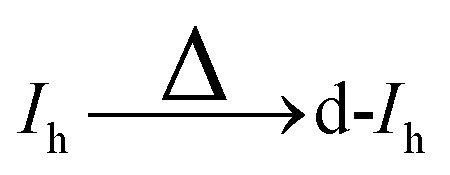	4.36 × 10^−2^	3.08 × 10^−1^	3.68 × 10^−1^	3.59 × 10^−1^
	8.6 × 10^−1^	2.4 × 10^4^	2.4 × 10^5^	1.7 × 10^5^

a Au_55_-*O*_h_ transforms into Au_55_-*I*_h_ instead of d-*O*_h_.

How can this be explained? One possibility would be that the free energy differences between the saddle point and the initial minimum are at room temperature much smaller than the energy difference at zero temperature that we have used in our calculations. Since soft modes lower the free energy more than hard modes, we have studied the modes at the Au_55_-*I-D*_5h_ minimum and its saddle point toward the Au_55_-*I*_h_. In Fig. 8 and 9, we plot both the exact potential and its quadratic approximation along the vibrational modes. Atomic displacements are generated according to

3 **R**^displaced^_*i*_ = **R**^0^_*i*_ + *x***ν**_*i*_,

where **R**^0^_*i*_ denotes the initial atomic positions of atom *i*, ***ν*** is an vibrational mode vector and *x* is the displacement amplitude since4
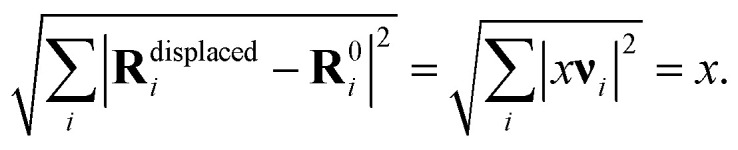


For each mode, atoms are displaced parallel and anti-parallel to the mode by an amplitude *x* to map out the local energy landscape around the reference structure. To assess whether the displacement amplitudes remain within the validity region of the harmonic approximation, the harmonic energy profile along the corresponding mode, given by 
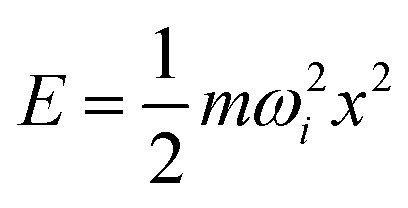
, is shown as a reference using dotted curves. *m* is the mass of a gold atom. By comparing Fig. 8 and 9, one sees that the saddle point has somewhat softer modes than the minimum, but the effect is too weak to bring down the theoretical predictions to the experimental values.

**Fig. 13:**
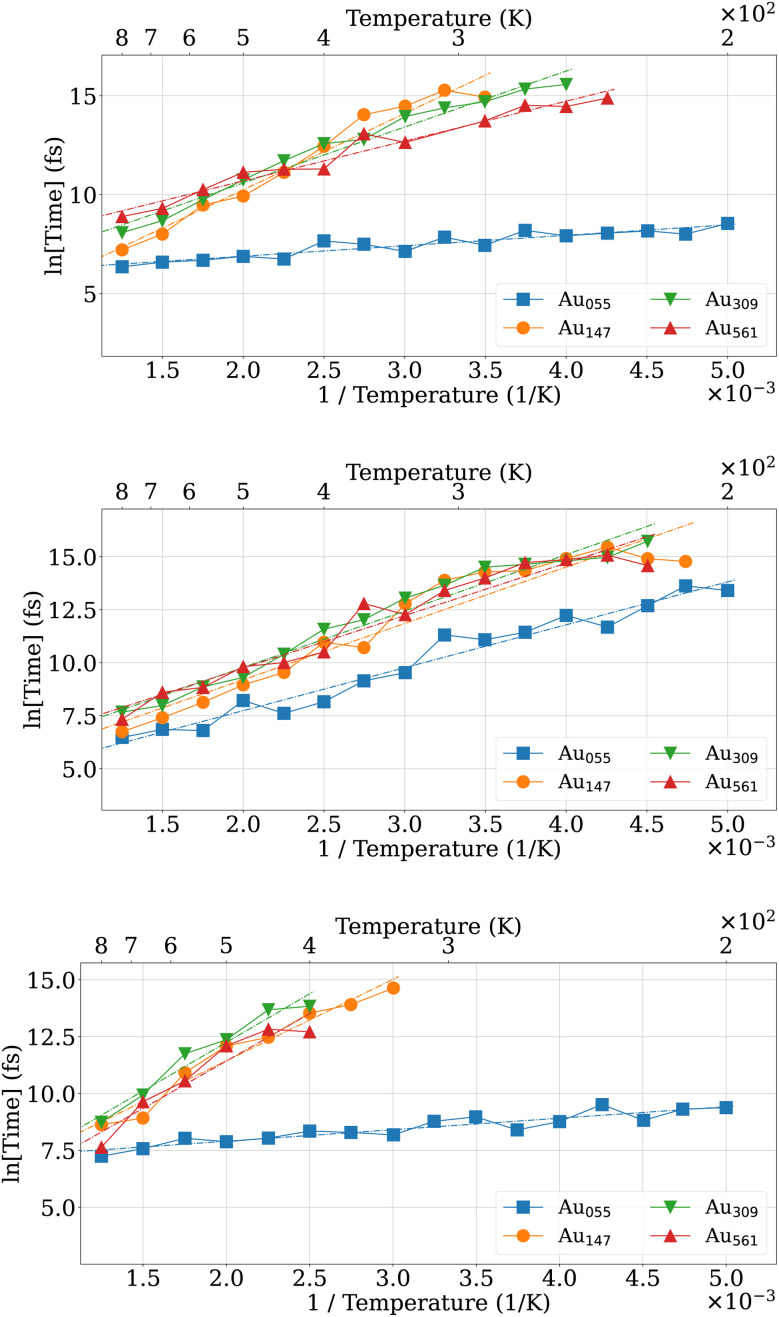
Van't Hoff plots for structural transformations of Au_55_, Au_147_, Au_309_ and Au_561_ starting from *O*_h_ (top), *I-D*_5h_ (middle) and *I*_h_ (bottom). The nearly identical slopes across different nanocluster sizes indicate that the transformation barriers are effectively size-independent.

The second possibility is that the systems in the experiment do not follow the symmetric jitterbug and slip-dislocation transformations but undergo asymmetric transformations, which have, as we have seen, a much lower barrier. To check this hypothesis, we performed ordinary MD trajectories starting from *O*_h_, *I-D*_5h_, and *I*_h_. The trajectories were interrupted by local geometry relaxations every 50 time steps, *i.e.*, every 250 fs, to check if the trajectory had left its initial catchment basin. If this were the case, the MD was stopped, and the time was recorded. Performing this procedure at different temperatures provided us with the data necessary to make the Van't Hoff plot shown in Fig. 13.

For small nanoclusters such as Au_55_ and Au_147_, the barrier heights obtained from the Van't Hoff analysis show good agreement with the calculated barriers along high-symmetry transformation pathways. This consistency indicates that the transformation kinetics in this size range are close to the high-symmetry transformations and well described by classical transition state theory.

For large nanoclusters, eqn (2) together with the barrier heights from Table 4, predicts transformation times for high-symmetry transformations that are longer than the age of the universe. Fig. 13 shows, however, that the effective barrier heights extracted from the Van't Hoff plots are nearly independent of nanocluster size, which is in agreement with experiments and COMPASS calculations. This implies that the larger nanoclusters never follow the symmetric jitterbug and slip-dislocation transformations. Instead, they follow asymmetric transformation pathways that consist of a series of localized transformation steps over low-energy barriers. One such step is shown in Fig. 15 for all nanocluster sizes. The localized nature becomes clearly visible by comparing the atomic displacements of these transformations with the ones shown in Fig. 15 and S8.

**Fig. 14:**
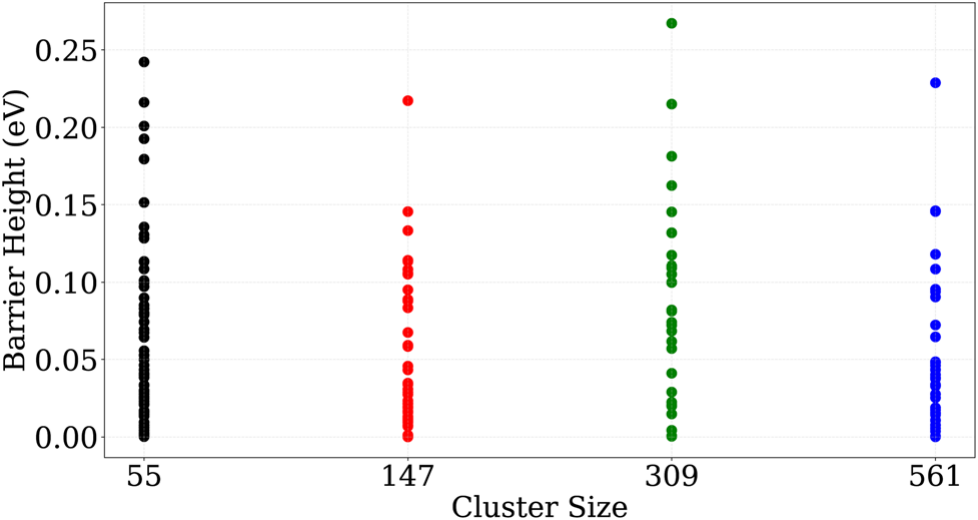
Barrier heights for the d-*I*_h_ → d-*I*_h_ transformation for Au_55_, Au_147_, Au_309_, and Au_561_.

The size-independent nature of these asymmetric rearrangements is further illustrated in Fig. 14. Across Au_55_, Au_147_, Au_309_, and Au_561_, the distribution of barrier heights remains remarkably similar. The large number of nearly degenerate d-*I*_h_ structures separated by low barriers indicates that large regions in the configuration space are accessible in the *I*_h_ funnel. Since the transformation times are, in most cases, much shorter than the time resolution of the experimental apparatus^6^ used to investigate the structure of these nanoclusters, experimentally measured properties will, in general, be averages over several distorted structures.

**Fig. 15:**
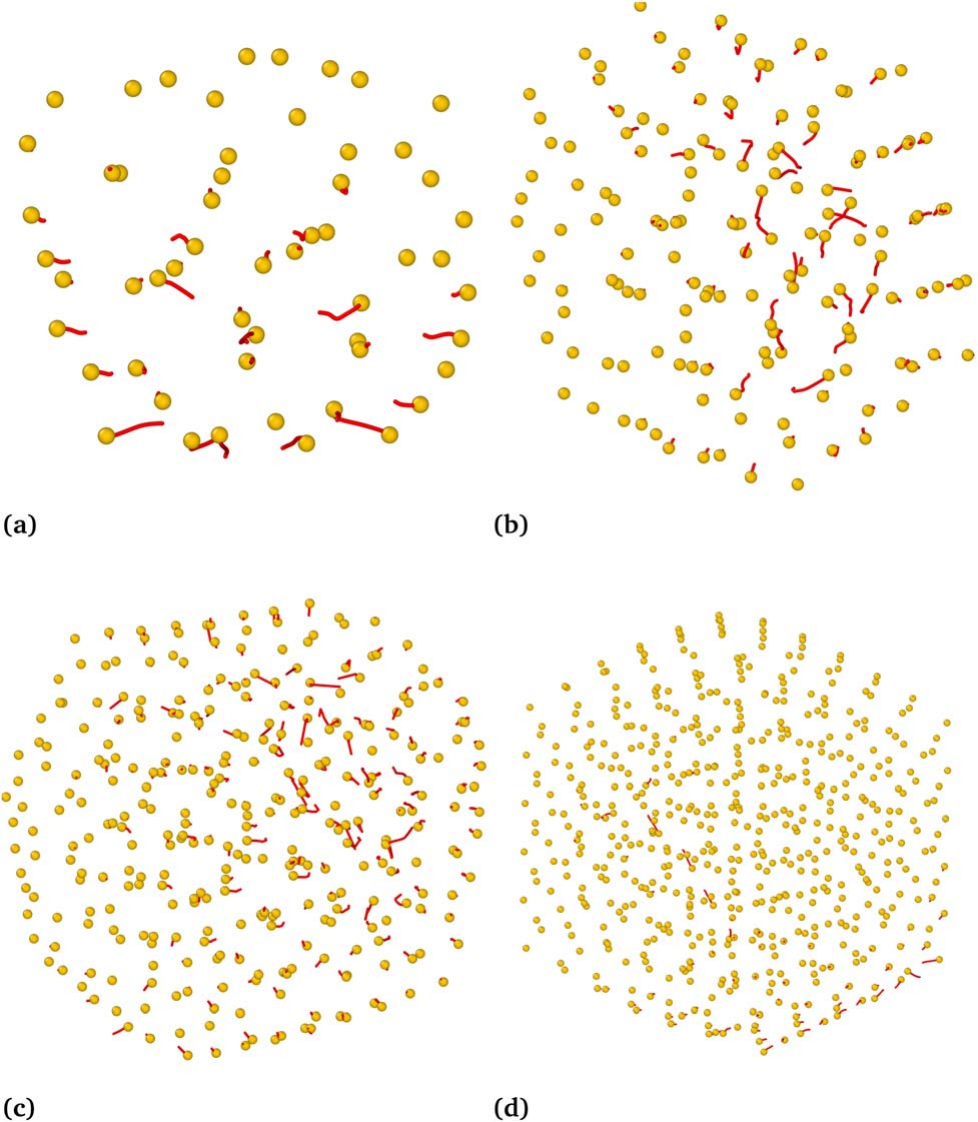
Localized displacement of atoms when transferring from one d-*I*_h_ structure to another d-*I*_h_ for Au_55_ (a), Au_147_ (b), Au_309_ (c) and Au_561_ (d).

## Conclusions

In this work, we first fully mapped out the transformation pathways between *I*_h_, *I-D*_5h_, and *O*_h_ gold nanoclusters with 55, 147, 309, and 561 atoms using a highly accurate machine learned interatomic potential. We find that these transformations are concerted movements of all atoms and that they proceed along the softest vibrational modes of the nanocluster. The atoms travel only relatively small distances during these transformations. The transformations are the atomistic analogues of the jitterbug and slip-dislocation mechanisms of elastic bodies. The height of the barrier of these transformations increases with increasing system size to values that make the transformation impossible on any experimental time scale. Hence, these symmetric transformation pathways cannot be the relevant ones in nature. We found numerous novel d-*I*_h_ structures that are much lower in energy than the *I*_h_, as well as low-lying distorted *I-D*_5h_ and *O*_h_ structures. We found that the barriers of transformations among these distorted structures are much lower than the barriers between the high-symmetry structures. This is because these transformations involve typically only a subset of atoms within a small region of the nanocluster. As a consequence, they can be overcome on relatively short time scales. We therefore postulate that the experimentally observed transitions between *I*_h_, *I-D*_5h_, and *O*_h_ shapes are not transitions between the high-symmetry shapes but among distorted-type structures.

## Conflicts of interest

There are no conflicts to declare.

## Supplementary Material

NA-OLF-D6NA00012F-s001

## Data Availability

The coordinate files for all structures and transformations reported in this article have been deposited and are provided in the accompanying zipped archive. Further information regarding the data can be obtained by contacting the corresponding author *via* email. Supplementary information (SI) is available. See DOI: https://doi.org/10.1039/d6na00012f.
