# Supplementary material for: The transformation mechanisms among cuboctahedra, Ino's decahedra and icosahedra structures of magic-size gold nanoclusters
Source: Nanoscale Adv. 2026 May 19. Online ahead of print. doi: 10.1039/d6na00012f (PMC13185000; doi:10.1039/d6na00012f)
Supplement: NA-OLF-D6NA00012F-s001 [file NA-OLF-D6NA00012F-s001.zip › SupportingInformation/SI.pdf]

Supporting Information:

The transformation mechanisms among cuboctahedra, Ino's decahedra and icosahedra structures of magic-size gold nanoclusters

Ehsan Rahmatizad Khajehpasha, Mohammad Ismaeil Safa, Nasrin Eyvazi
Marco Krummenacher, Stefan Goedecker

S1 Trainig Dataset

The training dataset was generated iteratively over five MH iterations. After each iteration, local minima together with high-energy structures on the MLIP PES were selected. Their energies and forces were computed using DFT with the settings listed in table SI. The MLIP model was then fine-tuned and used in the subsequent MH iteration. This procedure was repeated until a reliable MLIP was obtained. The energy and size distributions of the final dataset are shown in figures S1 and S2, respectively. Figure S3 depicts the energy correlation for nanoclusters of magic sizes that were excluded from the training dataset. Although only nanoclusters containing up to 90 atoms were included during training, the MLIP successfully extrapolates the PES to larger nanoclusters, achieving a root mean square error of ≈ 5 meV/atom and a correlation of 99.7% with the reference energies.

Table SI INCAR parameters used for a non-spin-polarized DFT calculation of Au

Tag	Value
PREC	Accurate
ISPIN	1
LREAL	Auto
ROPT	1E-4 1E-4 1E-4
ALGO	Fast
ISIF	0
ISYM	0
ISMEAR	0
SIGMA	0.05
POTIM	0.04
EDIFF	1×10^{-6}
GGA	PE #PBE
NSW	0
ENCUT	250
ENAUG	800

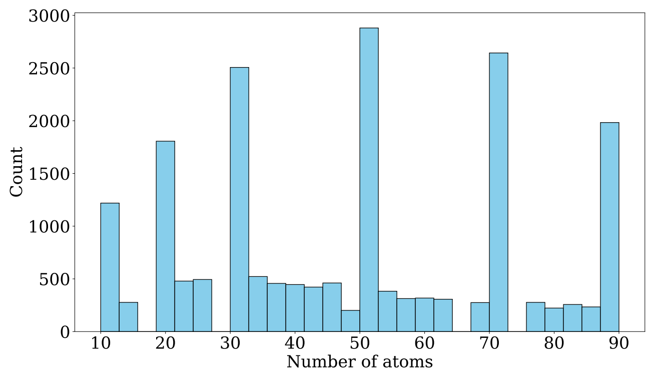

Fig. S1 Histogram for the size of structures in the training dataset.

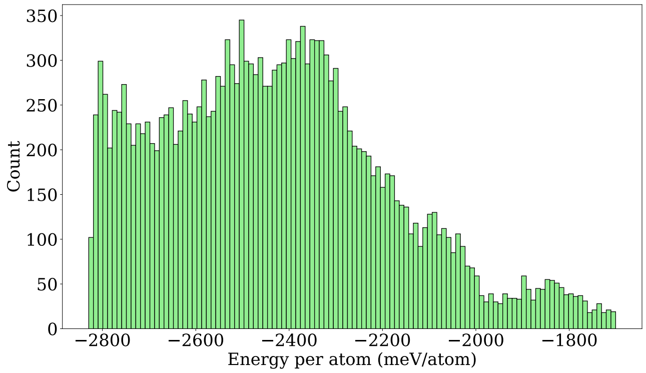

Fig. S2 Histogram for the energy of structures in the training dataset

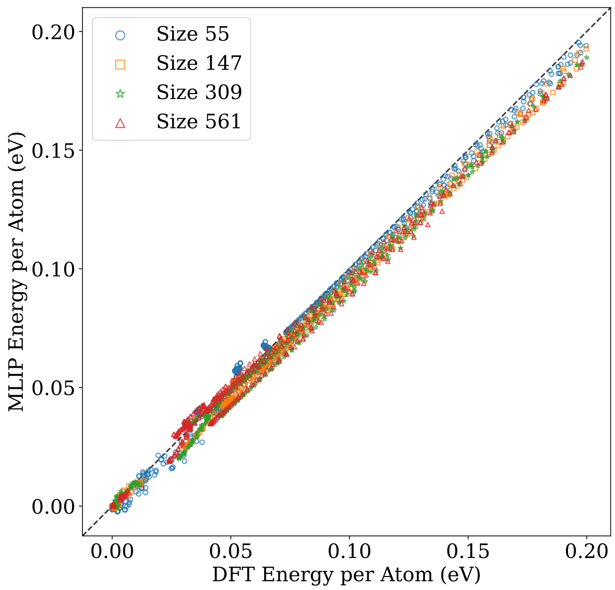

Fig. S3 Correlation of the energies calculated by the MLIP for the structures excluded from the training dataset of the MLIP.

S2 Saddle Point Search

Figure S4 shows an example of Python code for biased MH, with biasing weights listed in table SII for different transformations and sizes. The high-symmetry transformation pathways from biased MH were further optimized using the parameters in table SIII.

To assess the accuracy of the MLIP in describing energies and forces along transformation pathways, VASP NEB calculations were performed for Au_{55} and Au_{147} using the settings in table SIV, providing DFT-level validation of the predicted pathways and energy barriers.

While NEB captured high-symmetry transformations, it failed to locate saddle points for asymmetric transformations; these were instead obtained using COMPASS with the parameters in table SV.

```
from ase.io import read
from nequip.ase import NequIPCalculator
from minimahopping.minhop import Minimahopping
from biascalc.laplace_calculator
                      import LaplaceCalculator

target_configuration = read(
    'TargetStr.extxyz', format='extxyz'
)
configuration = read(
    'InputStr.extxyz', format='extxyz'
)
calculator = NequIPCalculator.from_deployed_model(
    model_path="b1_l1_r55n3f48.pth",
    device="cuda",
    species_to_type_name={"Au": "Au"},
)
bias_calculator = (
    LaplaceCalculator(
        target_configuration,
        bias=float('WBIAS')
    ).add_calculator(calculator)
)

configuration.calc = bias_calculator
with Minimahopping(
    configuration,
    md_calculator=bias_calculator,
    T0=300,
    dt0=1e-3,
    mdmin=1,
    n_soft=20,
    soften_positions=5e-2,
    fingerprint_threshold=0.1
) as mh:
    mh(totalsteps=500)
```

Fig. S4 Example Python code for performing biased MH with an MLIP and Laplace fingerprint biasing within the ASE framework.

Table SII Bias weight (ω in biased MH for the high-symmetry transformations

Transformation	Au_{55}	Au_{147}	Au_{309}	Au_{561}
$O_h \rightarrow I_h$	0.4	1.3	1.1	1.0
I-$D_{5h} \rightarrow I_h$	2.2	2.1	1.6	1.4
$I_h \rightarrow O_h$	6.2	3.9	2.7	1.8
$I_h \rightarrow$ I-D_{5h}	6.2	3.9	2.8	2.0

Table SIII Parameters used for the nudged elastic band calculation with MLIP for 21 images, optimized using the FIRE algorithm

Component	Parameter	Value
NEB	`climb`	True
NEB	Spring constant k	3.0
NEB	`allow_shared_calculator`	True
NEB	`remove_rotation_and_translation`	True
FIRE	`dtmax`	0.01
FIRE	`maxstep`	0.01
FIRE	`fmax`	0.005 #eV/Å

Table SIV INCAR parameters used for the nudged elastic band calculation of Au. The higher spring constant value compared to MLIP nudged elastic band calculation (table SIII) is due to a lower number of images

Tag	Value
PREC	Accurate
ISPIN	1
LREAL	Auto
ROPT	1E-4 1E-4 1E-4
LPLANE	.TRUE.
ALGO	Fast
NELM	300
NELMIN	5
EDIFF	1×10^{-8} #eV
ISMEAR	0
SIGMA	0.05 eV
ISYM	0
GGA	PE #(PBE)
ENCUT	300 eV
ENAUG	900 eV
IBRION	1
POTIM	0.5
EDIFFG	−0.010 #eV/Å
ISIF	0
NSW	1000
IMAGES	7
SPRING	−5.0
LCLIMB	.TRUE.

Table SV Input parameters of COMPASS calculations

Parameter	Value
minimize_input	True
nsteps	10000
nloops	200
forcetol	0.005
forcetolperdistance	0.005
distancestepmax	1.0
distancestepmin	0.001
targetdistancesaddle	0.1
targetdistanceother	0.2
energydifferencethreshold	0.005
nsplitdouble	2
nsplitsingle	1
alpha0	0.001
trustradius	0.05
trustedforcedifference	0.5
extracurv	False
saddle_cosp	0.7
nhistx	10
restart_level	2
restart_interval	1
basename	clog_
minimize_fmax	0.001
minimize_nsteps	4000
minimize_maxrise	0.005
parallel	False
incremental_projection	True
interpolation_idpp	False
scalar_pressure	0.0

S3 Ionized NEB of Au_{55}

To assess the effect of ionization on high-symmetry transformation pathways, we performed NEB calculations for Au_{55} using the FHI-aims code with an intermediate basis set. The NEB was relaxed to a force tolerance of ten meV/atom. The parameters used in these calculations are listed in table SVI, with the total charge adjusted according to the ionization state [1,2].

Table SVI FHI-aims parameters used for NEB calculations of ionized Au_{55}

Parameter	Value
Exchange-correlation (xc)	PBE
Total charge	Ionization state dependent
Spin treatment	none
Relativistic treatment	atomic_zora scalar
Compute forces	true
SCF energy convergence	1×10^{-6}
SCF force convergence	1×10^{-4}

Table SVII RMSD values (Å) for each image relative to the corresponding structure in the neutral Au_{55} high-symmetry transformation pathway.

	Image #	Au_{55}^{-2}	Au_{55}^{-1}	Au_{55}^{+1}	Au_{55}^{+2}
$O_h \rightarrow I_h$	1	0.024	0.008	0.008	0.016
	2	0.033	0.009	0.011	0.016
	3	0.050	0.011	0.018	0.015
	4	0.053	0.015	0.027	0.025
	5	0.039	0.013	0.023	0.019
	6	0.025	0.009	0.016	0.013
	7	0.014	0.006	0.008	0.009
	8	0.008	0.004	0.004	0.008
I-$D_{5h} \rightarrow I_h$	1	0.056	0.026	0.030	0.022
	2	0.058	0.029	0.030	0.027
	3	0.054	0.033	0.029	0.030
	4	0.057	0.025	0.028	0.029
	5	0.053	0.024	0.030	0.024
	6	0.051	0.024	0.026	0.019
	7	0.049	0.023	0.023	0.013
	8	0.008	0.004	0.004	0.008

Table SVIII Minimum and maximum atomic displacements in Å during Au_{55} high-symmetry transformations calculated with FHI-Aims for different ionization states, excluding the innermost atom. The values in parentheses are from table 2, i.e. were obtained from MLIP calculations.

Transformation	Ionization State	Min (Å)	Max (Å)
	-2	0.6	1.3
	-1	0.6	1.3
$O_h \rightarrow I_h$	0	0.6 (0.4)	1.3 (1.3)
	+1	0.6	1.3
	+2	0.7	1.3
	-2	0.1	1.6
	-1	0.1	1.6
I-$D_{5h} \rightarrow I_h$	0	0.1 (0.1)	1.6 (1.5)
	+1	0.1	1.6
	+2	0.1	1.6

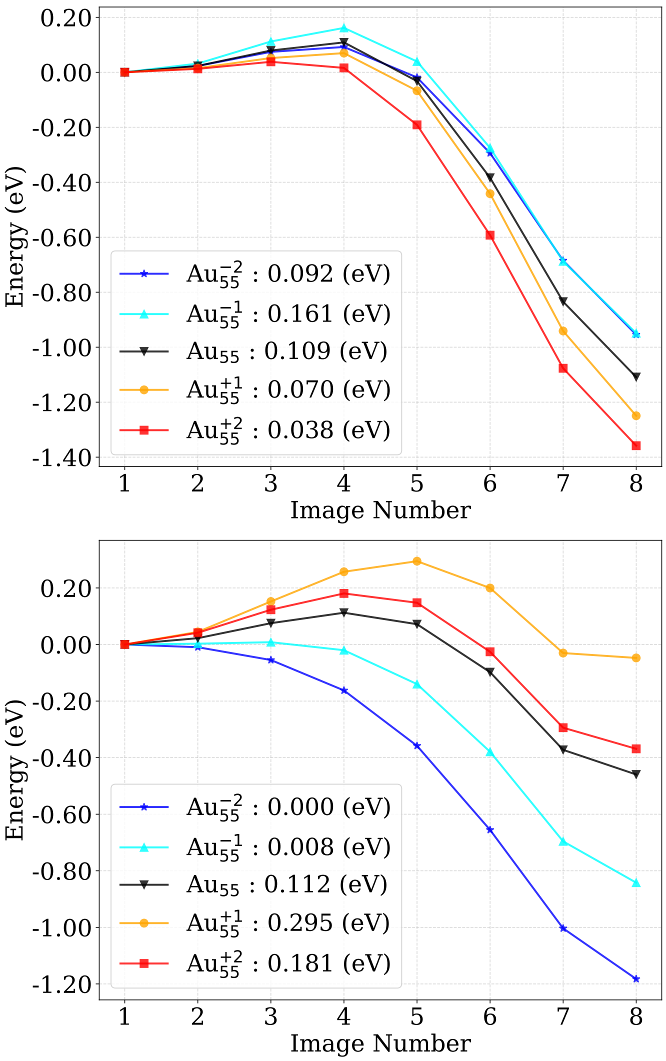

Fig. S5 The energy of NEB pathways calculated using FHI-aims for different ionization states of Au_{55} for the $O_h \rightarrow I_h$ (top) and I-$D_{5h} \rightarrow I_h$ (bottom) transformations. The values in the legend indicate the corresponding barrier heights for each ionization state.

Table SVII summarizes the RMSD of each image along the high-symmetry transformation pathway for different ionization states relative to neutral Au_{55}. Table SVIII presents the corresponding atomic displacement amplitudes during these transformations. Notably, the RMSD values are approximately an order of magnitude smaller than the minimum atomic displacements observed, demonstrating in this way that the transformation pathway is quite insensitive to the ionization state. Further comparison with figure S5 shows that, while ionization does not significantly alter the geometries along the high-symmetry transformation pathways, it does affect the associated barrier heights.

S4 Global Optimization

Table SIX presents the parameters used in MH to explore the PES in the pursuit of identifying the GM. Newly identified GM structures were further relaxed using VASP with the parameters listed in table SX.

Table SIX Parameters used for the Minima Hopping global optimization run

Parameter	Value
T_0	600
Δt_0	1×10^{-2}
$E_{\text{diff},0}$	0.1
Fingerprint threshold	0.005
Energy threshold	0.010
MD minimum steps	10
Softening steps (n_{soft})	100
$f_{\max}$	1×10^{-3}
MPI enabled	True
Total steps	4000

Table SX INCAR parameters used for a spin-polarized DFT calculation of gold with dipole corrections. To disable geometry optimization for single-point calculations, NSW was set to zero

Tag	Value
PREC	Accurate
ISPIN	2
LDIPOL	.TRUE.
IDIPOL	4
DIPOL	0.5 0.5 0.5
LREAL	Auto
ROPT	1E-4 1E-4 1E-4
LPLANE	.TRUE.
ALGO	Normal
NELM	1000
ISIF	0
ISYM	0
ISMEAR	0
SIGMA	0.0005
IBRION	2
POTIM	0.1
EDIFFG	-0.001
EDIFF	1×10^{-9}
GGA	PE
NSW	1000
ENCUT	300 #eV

S5 Energy Profiles Along Low-Frequency Mode

Figure S6 shows the energy profile of structures displaced along the lowest positive mode. The softness of these modes introduces significant numerical noise in vibrational analysis using the finite-difference method.

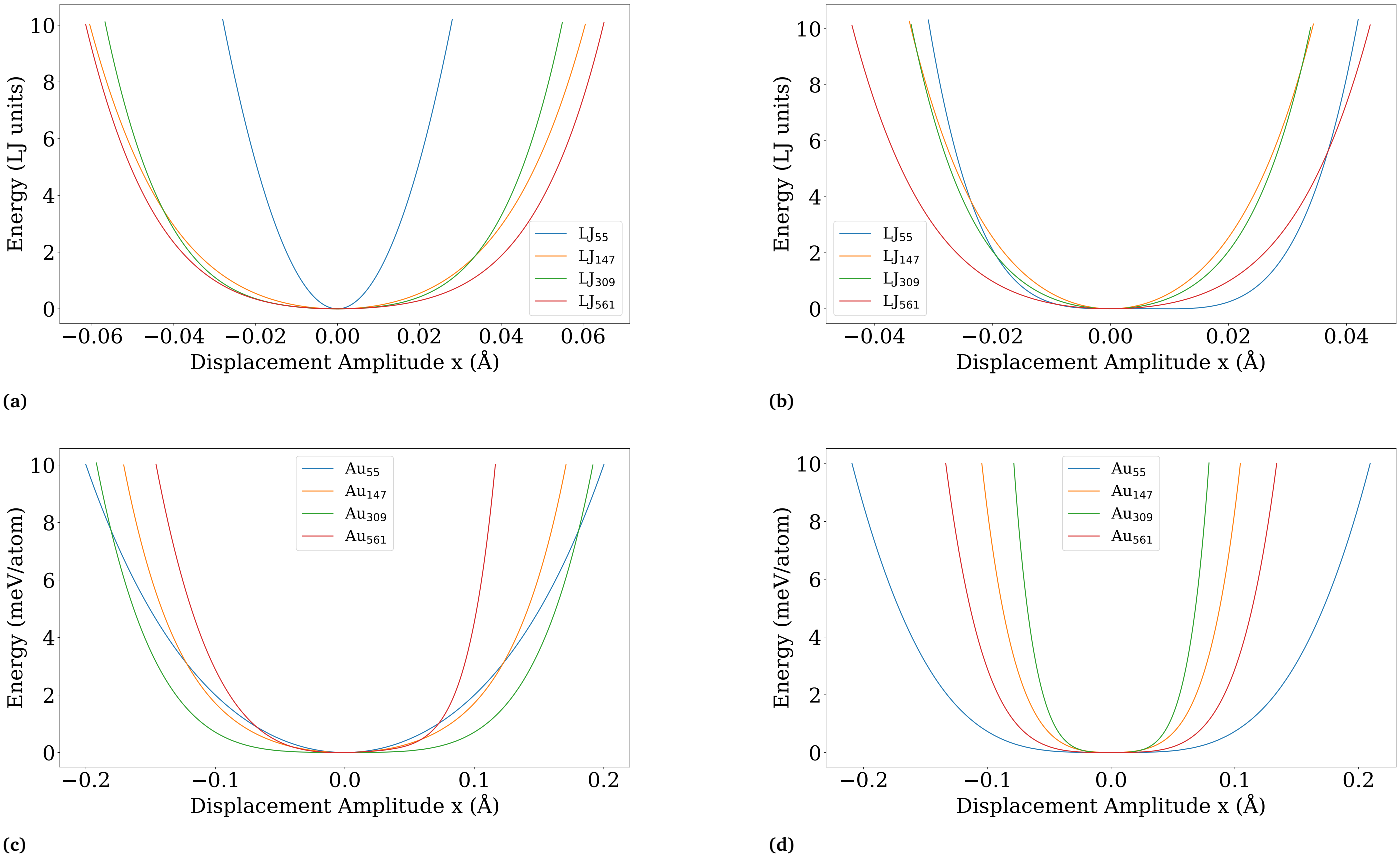

Fig. S6 The energy profile for the saddle points of LJ-O_h → LJ-I_h (a), LJ-I-D_{5h} → LJ-I_h (b), Au-O_h → Au-I_h (c), Au-I-D_{5h} → Au-I_h (d) transformations displaced parallel to the softest positive mode. The negative sign of the *displacement amplitude x* represents a displacement antiparallel to the softest positive mode.

S6 Role of Soft Modes in and asymmetric Transformations

Figure S7 illustrates the angles between the vector connecting the initial structure to the asymmetric transformation saddle point and the individual vibrational mode eigenvectors, thereby quantifying the contribution of each mode in initiating the transformation to the low-energy asymmetric saddle points. While high-symmetry transformation pathways are largely aligned only with the softest vibrational mode, asymmetric transformations involve contributions from multiple vibrational modes along the pathway to the saddle point, where soft modes play a dominant role. Since high-symmetry pathways and their dominance by the softest mode have already been discussed in section 3.2 , they are not revisited here.

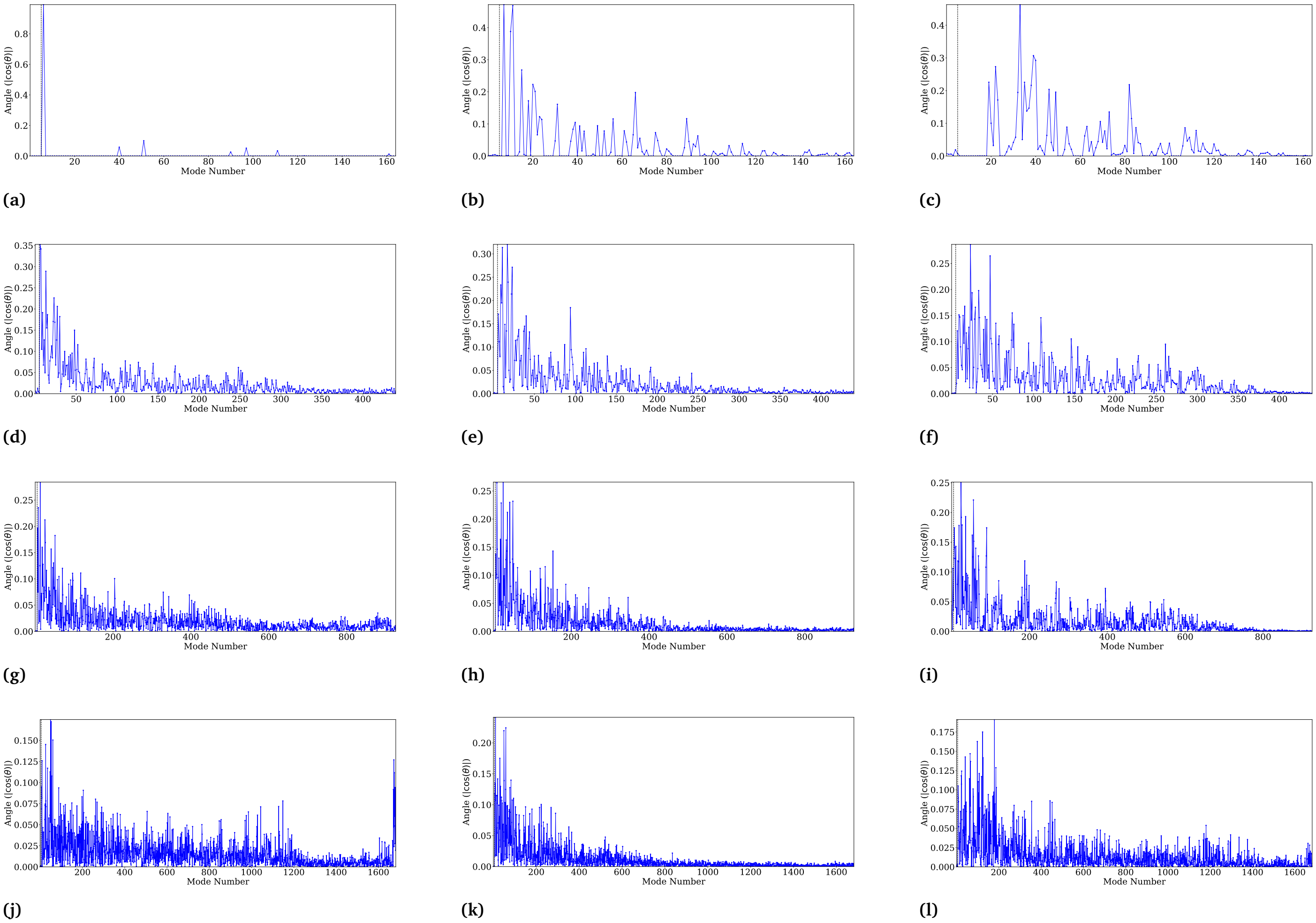

Fig. S7 The angles between the vector connecting the initial structure to the asymmetric transformation saddle point and each vibrational mode are shown. Columns correspond to the initial structures: O_h (left), I-D_{5h} (middle), and I_h (right). Rows correspond to nanocluster sizes: Au_{55} (first), Au_{147} (second), Au_{309} (third), and Au_{561} (fourth).

S7 Minimum Energy Pathways and Vibrational Modes

The minimum energy pathways for high-symmetry structural transformations of Au nanoclusters are shown in figure S8. The top row corresponds to the I-$D_{5h} \rightarrow I_h$ transformations of Au_{147}, Au_{309}, and Au_{561}, while the bottom row depicts the $O_h \rightarrow I_h$ transformations for the same nanocluster sizes. Each transformation pathway is accompanied by the corresponding softest vibrational mode, and the atomic coordinates for each step are provided in the **pathway** directory of the supplementary material for detailed visualization.

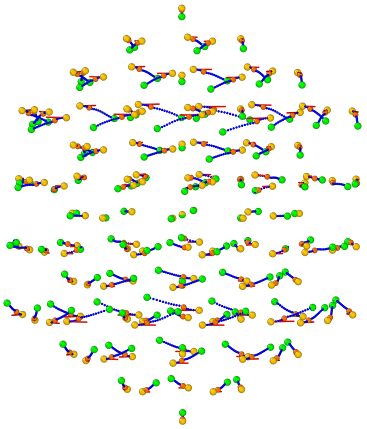

(a)

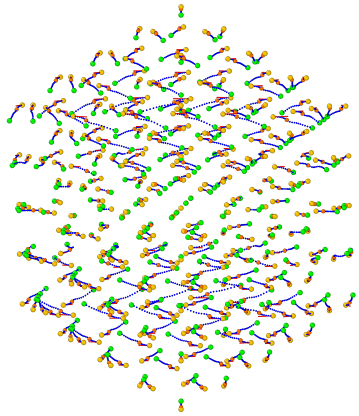

(b)

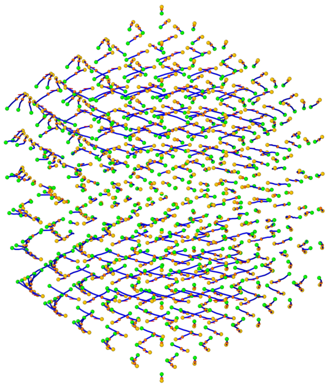

(c)

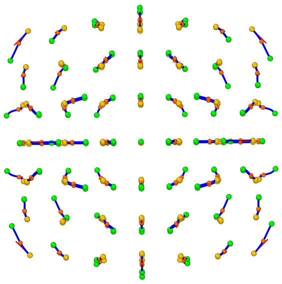

(d)

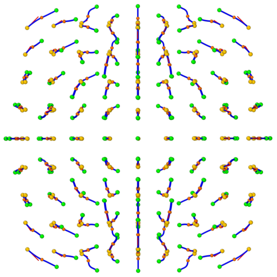

(e)

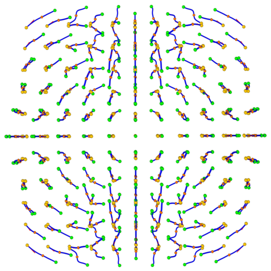

(f)

Fig. S8 High-symmetry transformation pathways of Au nanoclusters. Top row: side views of I-$D_{5h} \rightarrow I_h$ transformations for Au_{147} (a), Au_{309} (b), and Au_{561} (c). Bottom row: (100) views of $O_h \rightarrow I_h$ transformations for the same nanoclusters (d–f). Golden spheres represent initial structures, green spheres indicate the final I_h configuration, red lines show the softest vibrational mode, blue lines trace the NEB pathway, and orange highlights the saddle-point configuration.

Notes and references

1 V. Blum, R. Gehrke, F. Hanke, P. Havu, V. Havu, X. Ren, K. Reuter and M. Scheffler, *Computer Physics Communications*, 2009, **180**, 2175–2196.

2 A. Marek, V. Blum, R. Johanni, V. Havu, B. Lang, T. Auckenthaler, A. Heinecke, H.-J. Bungartz and H. Lederer, *Journal of Physics: Condensed Matter*, 2014, **26**, 213201.
