# Supplementary figures and images for: The transformation mechanisms among cuboctahedra, Ino's decahedra and icosahedra structures of magic-size gold nanoclusters
Source: Nanoscale Adv. 2026 May 19. Online ahead of print. doi: 10.1039/d6na00012f (PMC13185000; doi:10.1039/d6na00012f)

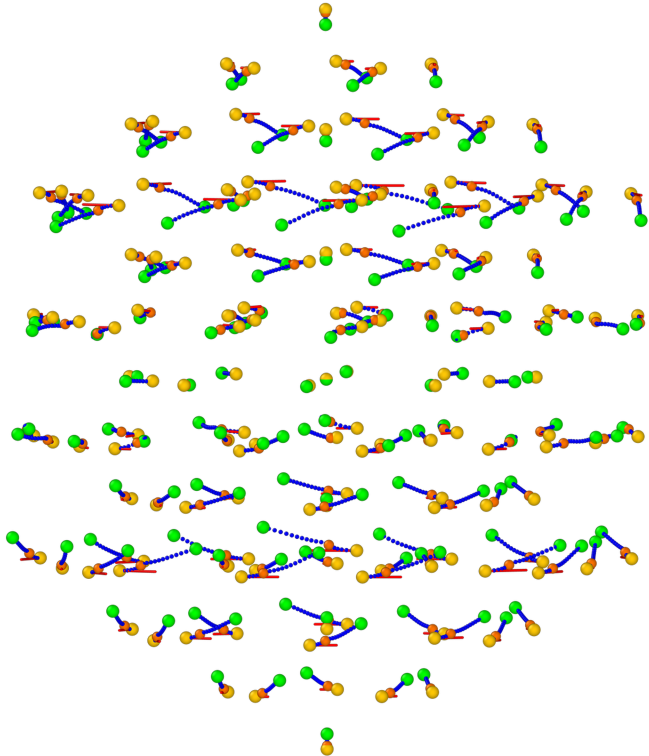

Supplement: NA-OLF-D6NA00012F-s001 [file NA-OLF-D6NA00012F-s001.zip › SupportingInformation/SI-Figures/147_DH_ICS_Side.pdf]

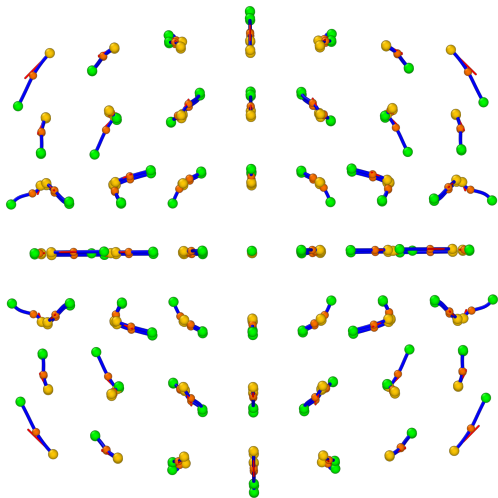

Supplement: NA-OLF-D6NA00012F-s001 [file NA-OLF-D6NA00012F-s001.zip › SupportingInformation/SI-Figures/147_FCC_ICS_100.pdf]

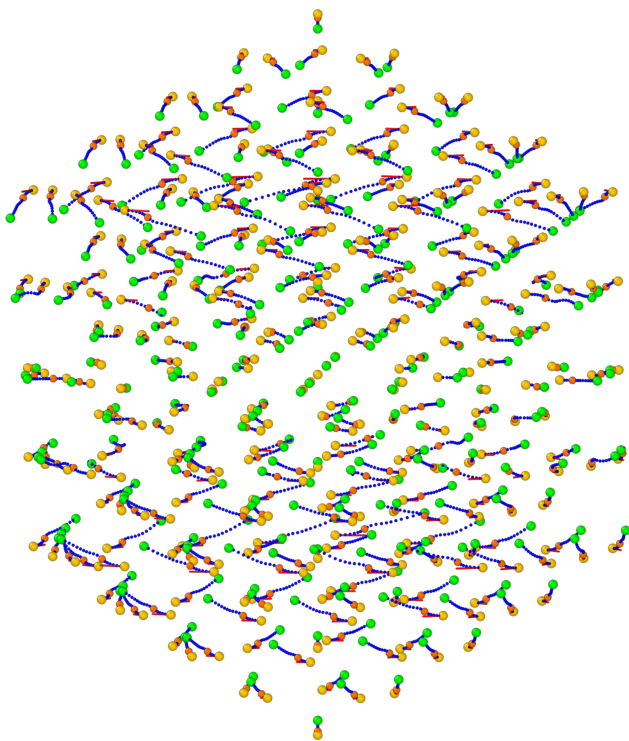

Supplement: NA-OLF-D6NA00012F-s001 [file NA-OLF-D6NA00012F-s001.zip › SupportingInformation/SI-Figures/309_DH_ICS_Side.pdf]

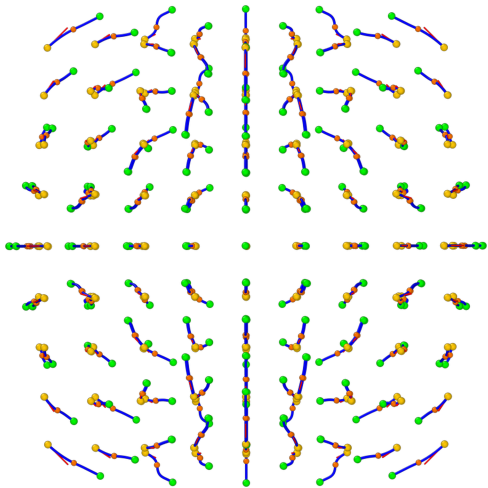

Supplement: NA-OLF-D6NA00012F-s001 [file NA-OLF-D6NA00012F-s001.zip › SupportingInformation/SI-Figures/309_FCC_ICS_100.pdf]

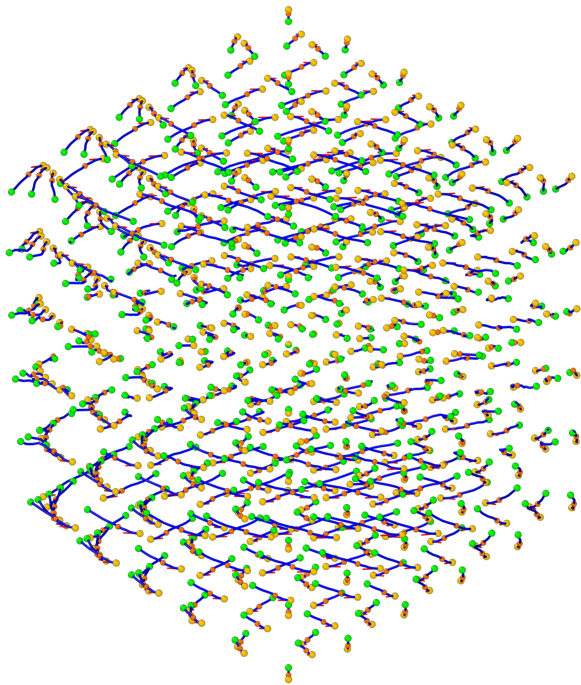

Supplement: NA-OLF-D6NA00012F-s001 [file NA-OLF-D6NA00012F-s001.zip › SupportingInformation/SI-Figures/561_DH_ICS_Side.pdf]

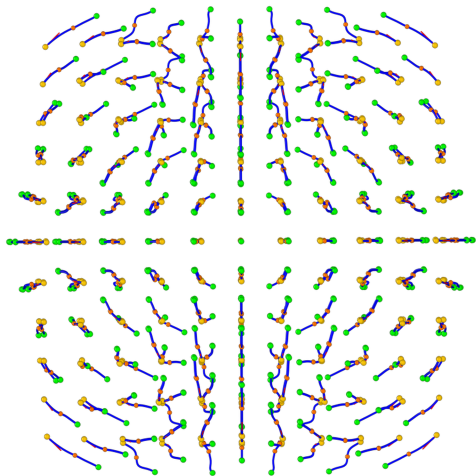

Supplement: NA-OLF-D6NA00012F-s001 [file NA-OLF-D6NA00012F-s001.zip › SupportingInformation/SI-Figures/561_FCC_ICS_100.pdf]

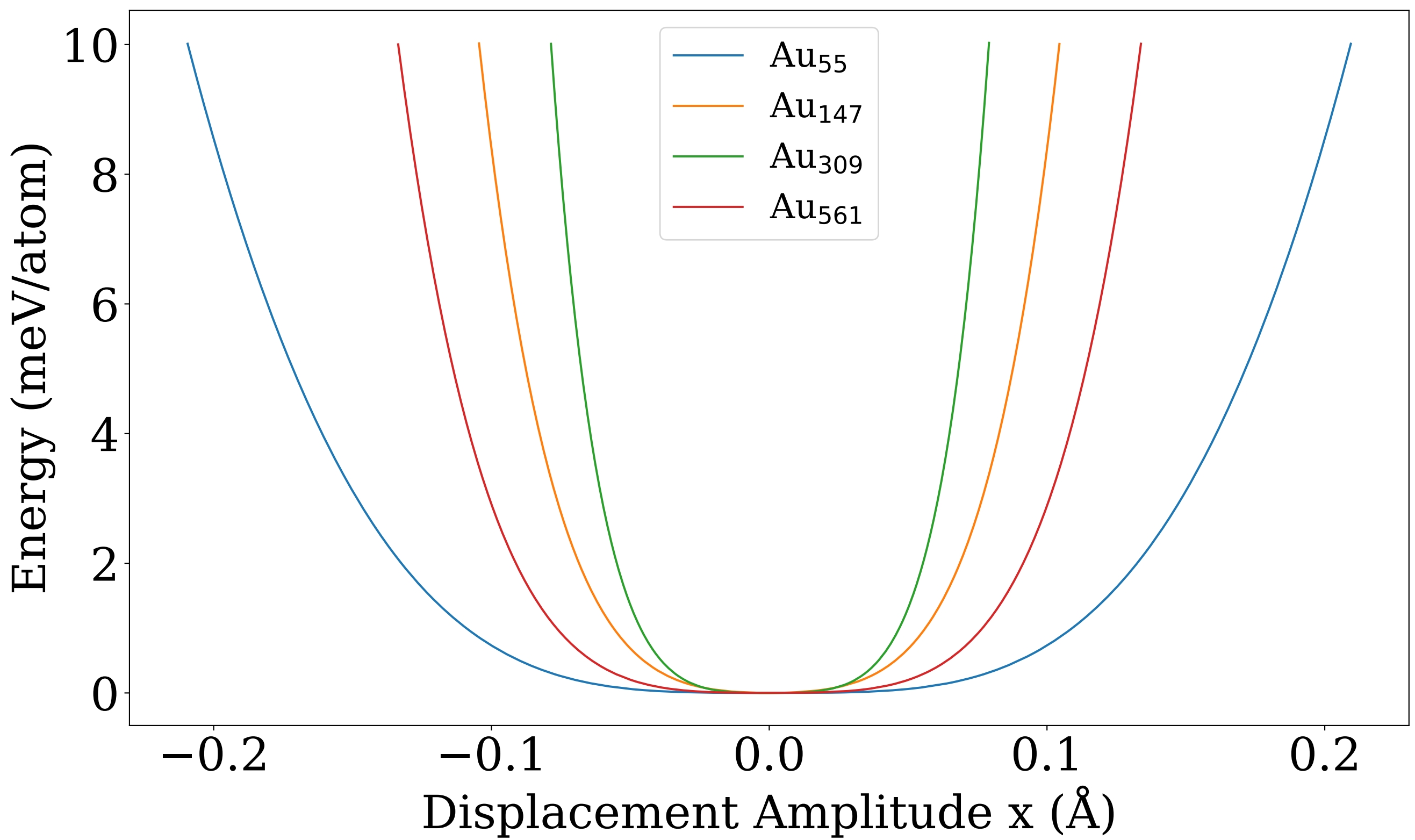

Supplement: NA-OLF-D6NA00012F-s001 [file NA-OLF-D6NA00012F-s001.zip › SupportingInformation/SI-Figures/All_DH_7_Au.pdf]

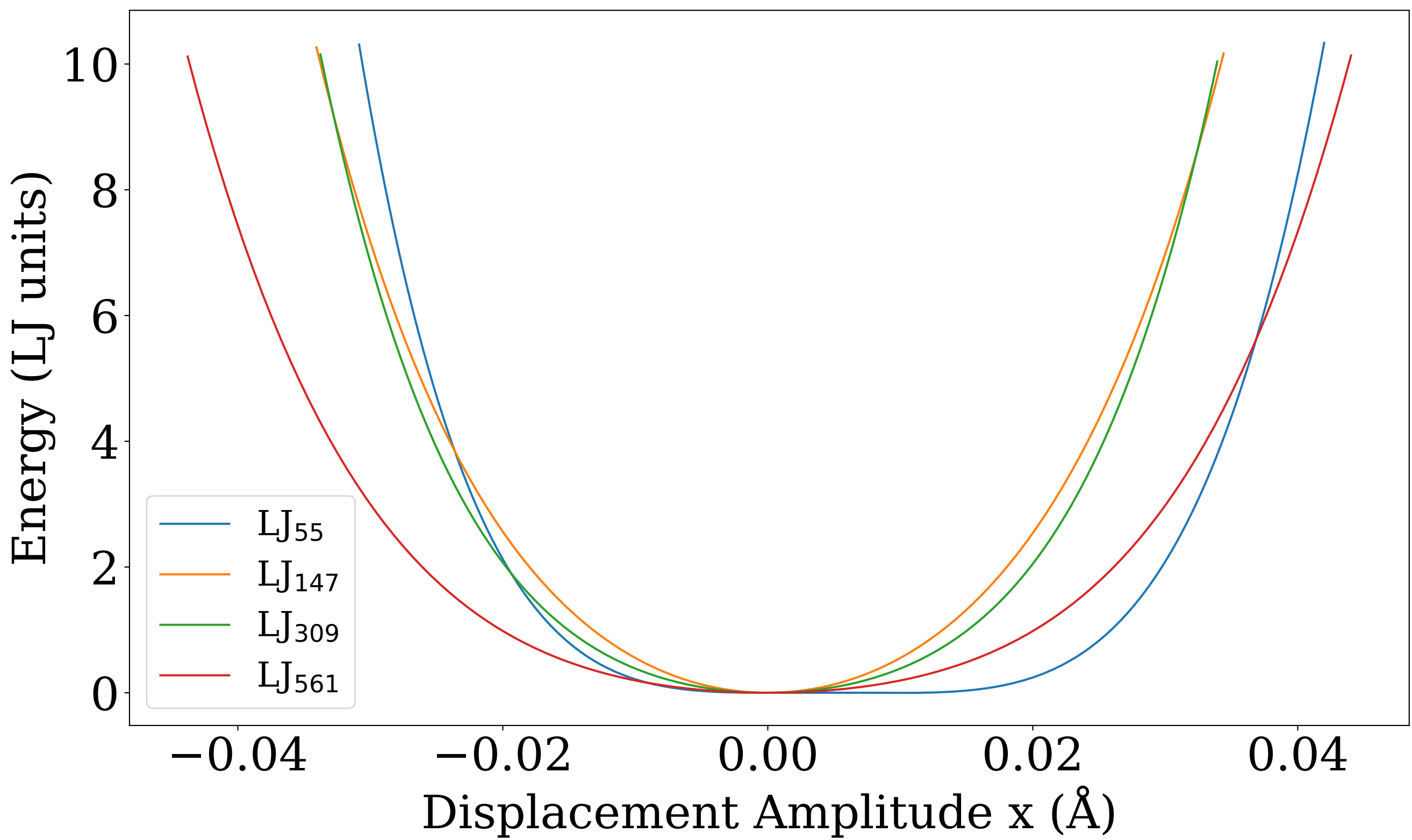

Supplement: NA-OLF-D6NA00012F-s001 [file NA-OLF-D6NA00012F-s001.zip › SupportingInformation/SI-Figures/All_DH_7_LJ.pdf]

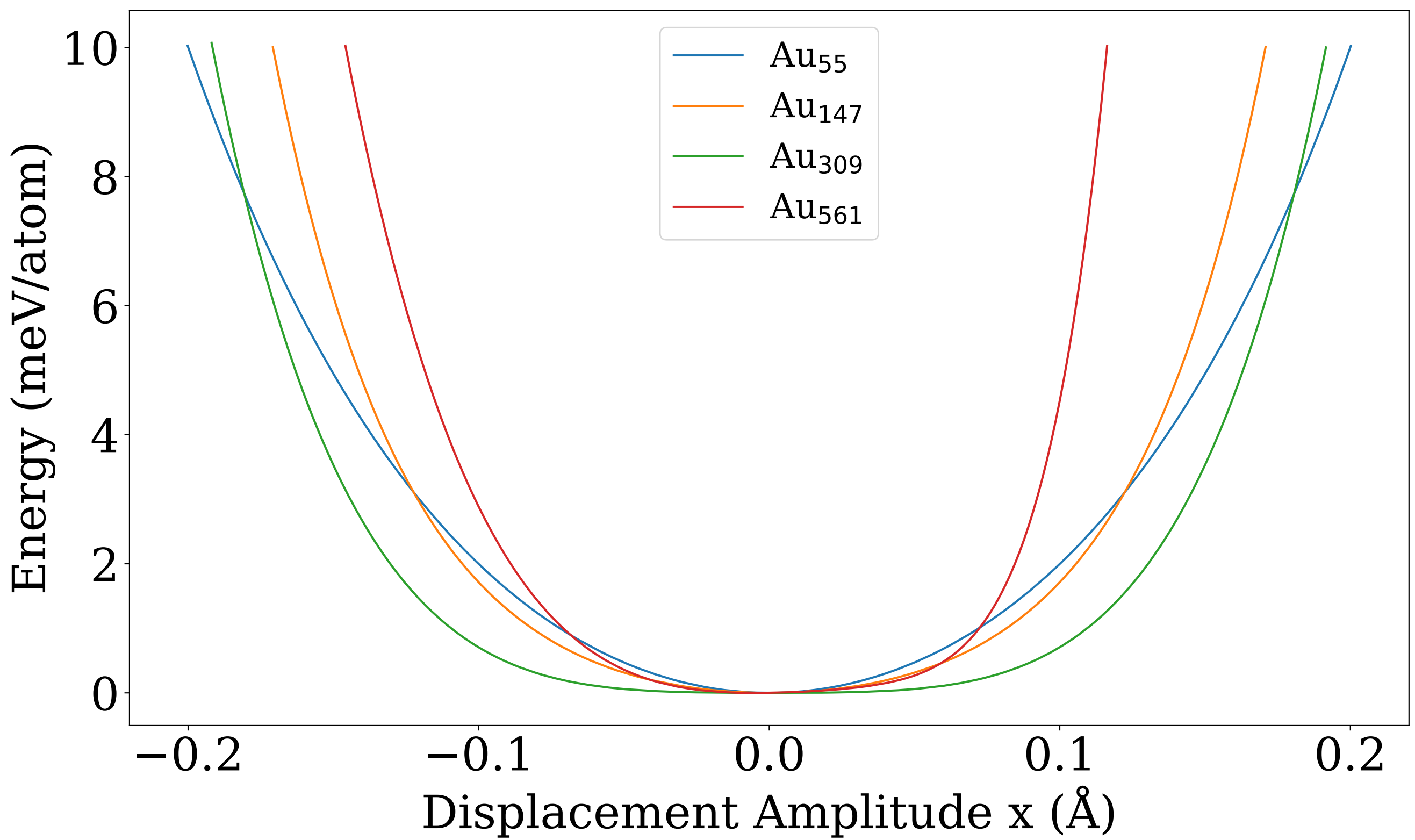

Supplement: NA-OLF-D6NA00012F-s001 [file NA-OLF-D6NA00012F-s001.zip › SupportingInformation/SI-Figures/All_FC_7_Au.pdf]

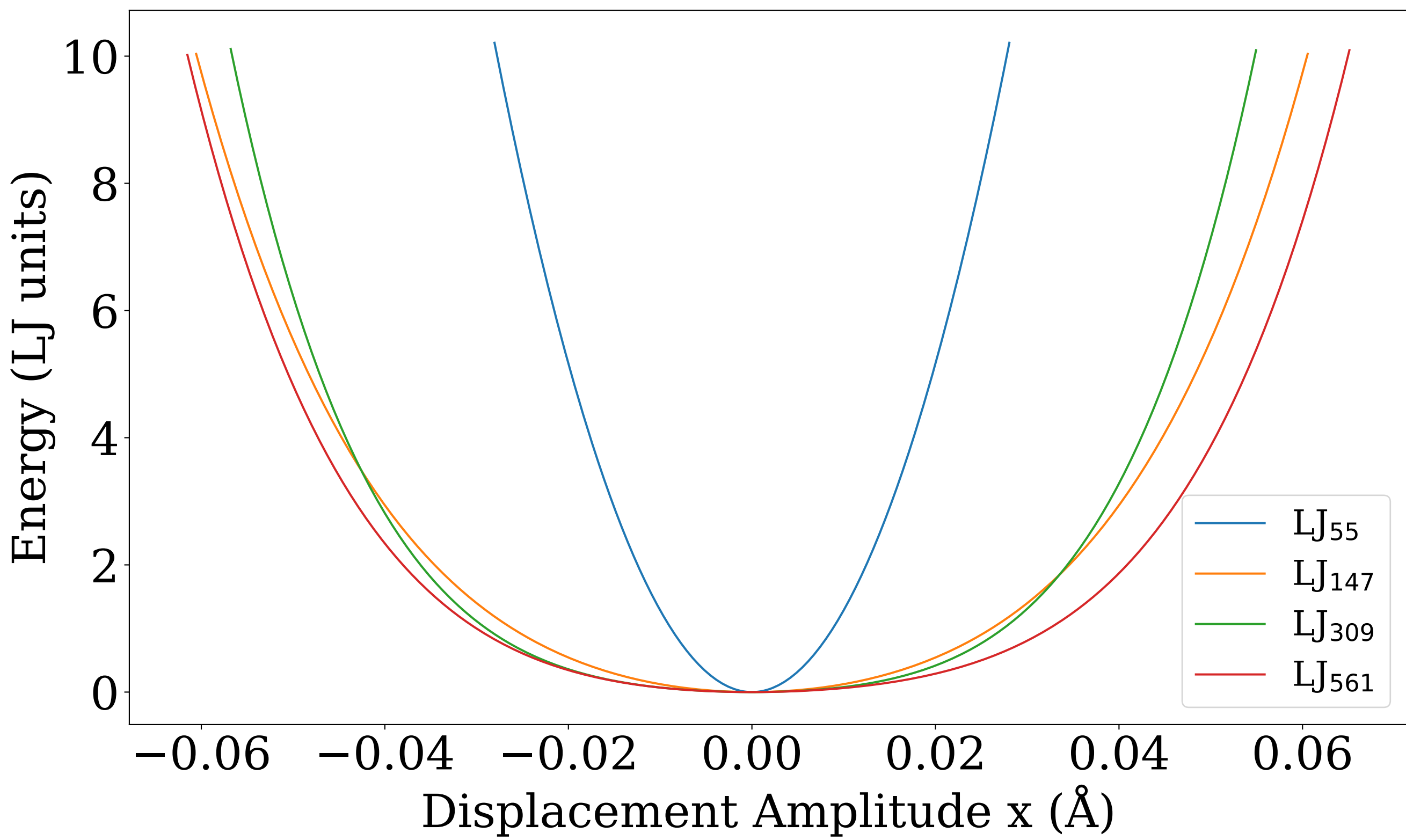

Supplement: NA-OLF-D6NA00012F-s001 [file NA-OLF-D6NA00012F-s001.zip › SupportingInformation/SI-Figures/All_FC_7_LJ.pdf]

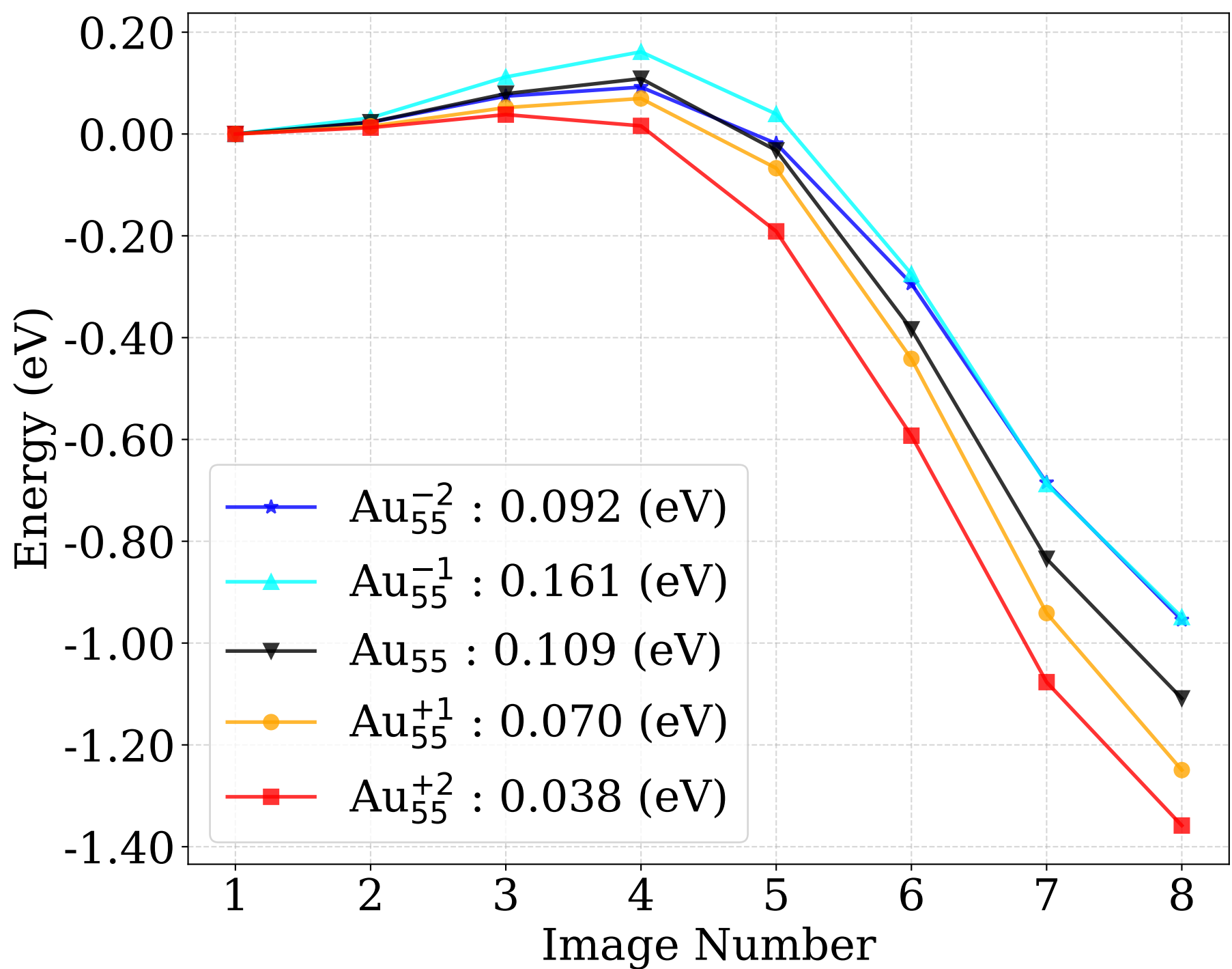

Supplement: NA-OLF-D6NA00012F-s001 [file NA-OLF-D6NA00012F-s001.zip › SupportingInformation/SI-Figures/Energy_Pathway_CO.pdf]

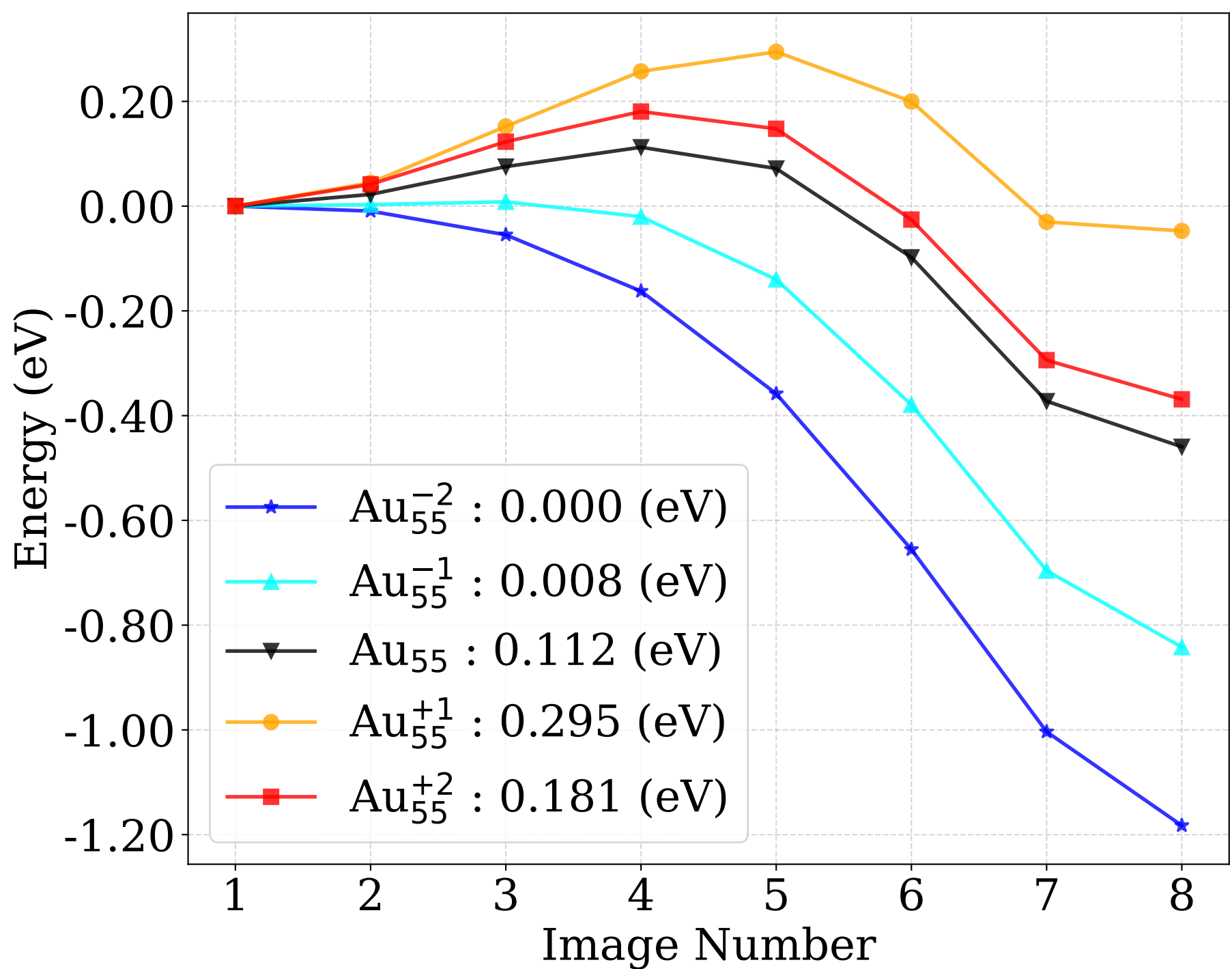

Supplement: NA-OLF-D6NA00012F-s001 [file NA-OLF-D6NA00012F-s001.zip › SupportingInformation/SI-Figures/Energy_Pathway_DH.pdf]

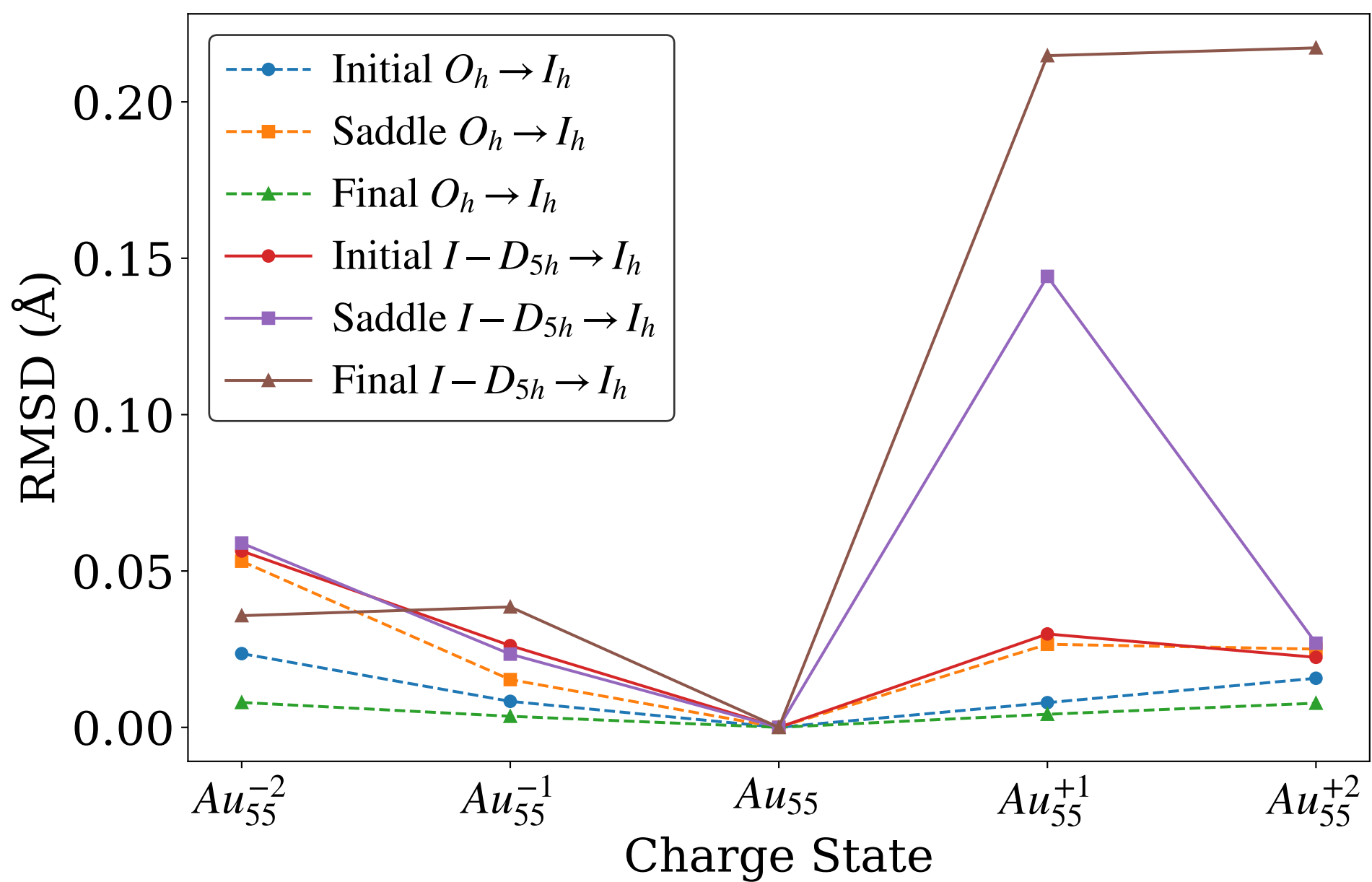

Supplement: NA-OLF-D6NA00012F-s001 [file NA-OLF-D6NA00012F-s001.zip › SupportingInformation/SI-Figures/RMSD.pdf]

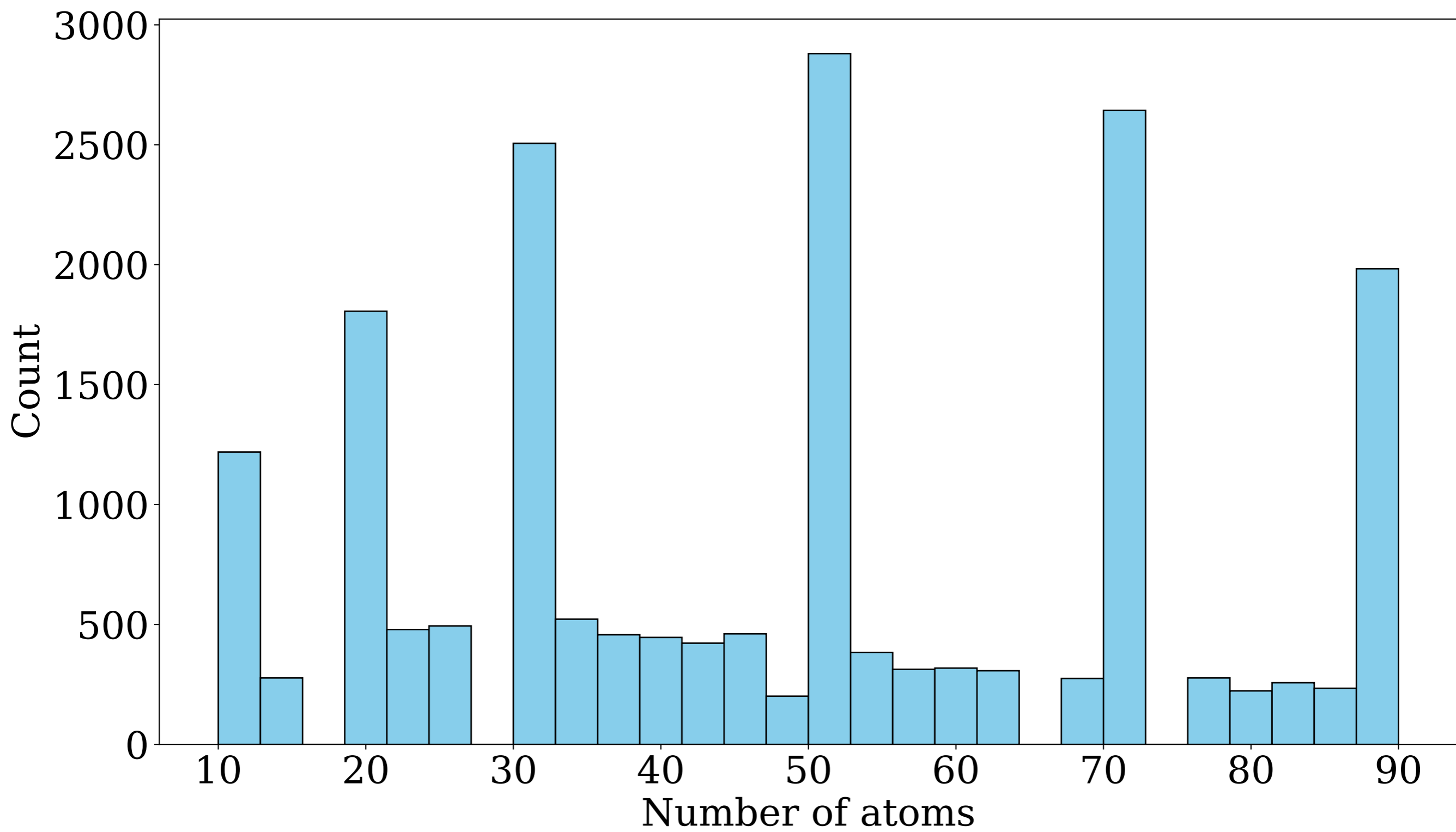

Supplement: NA-OLF-D6NA00012F-s001 [file NA-OLF-D6NA00012F-s001.zip › SupportingInformation/SI-Figures/count.pdf]

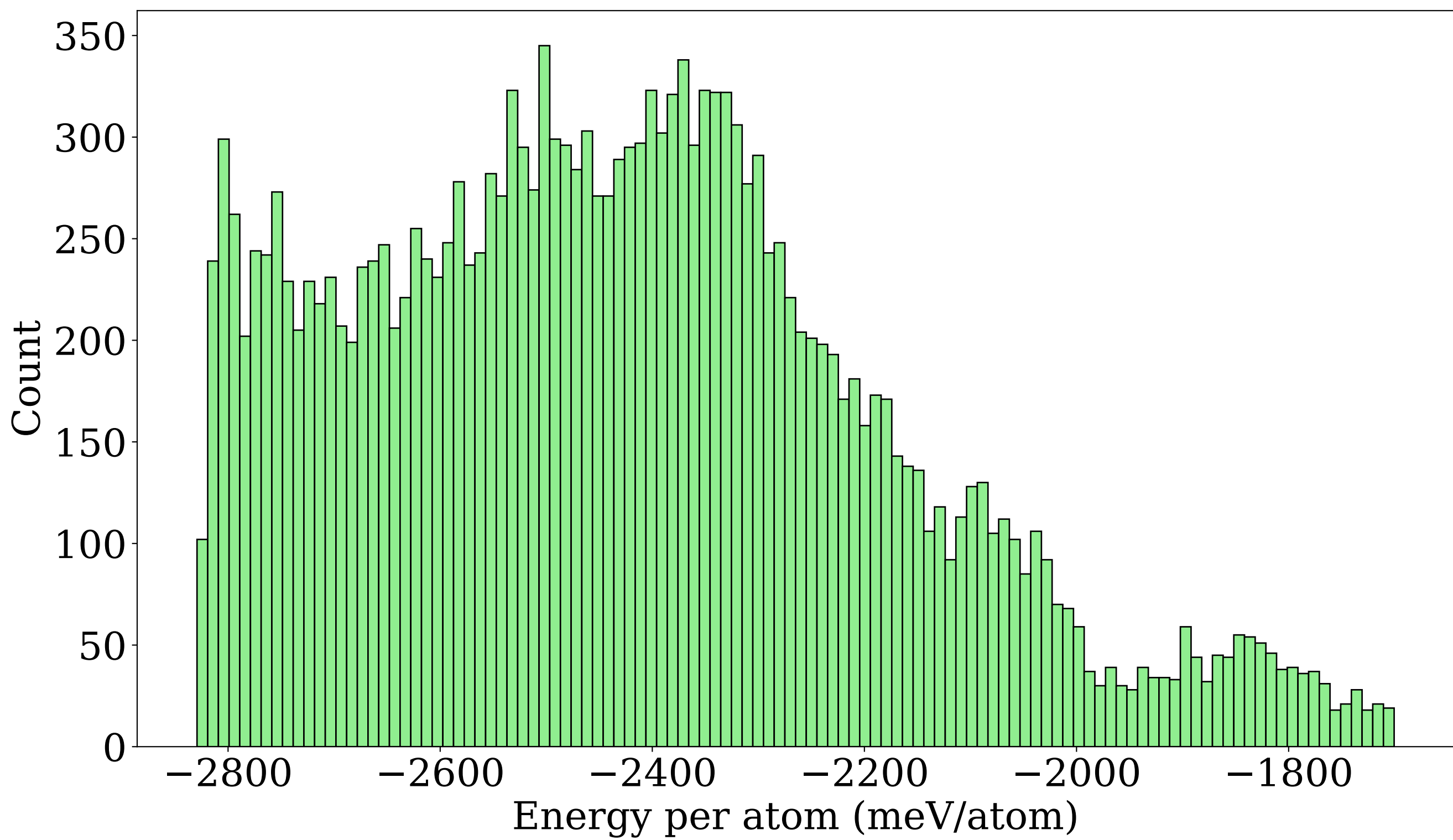

Supplement: NA-OLF-D6NA00012F-s001 [file NA-OLF-D6NA00012F-s001.zip › SupportingInformation/SI-Figures/eners.pdf]

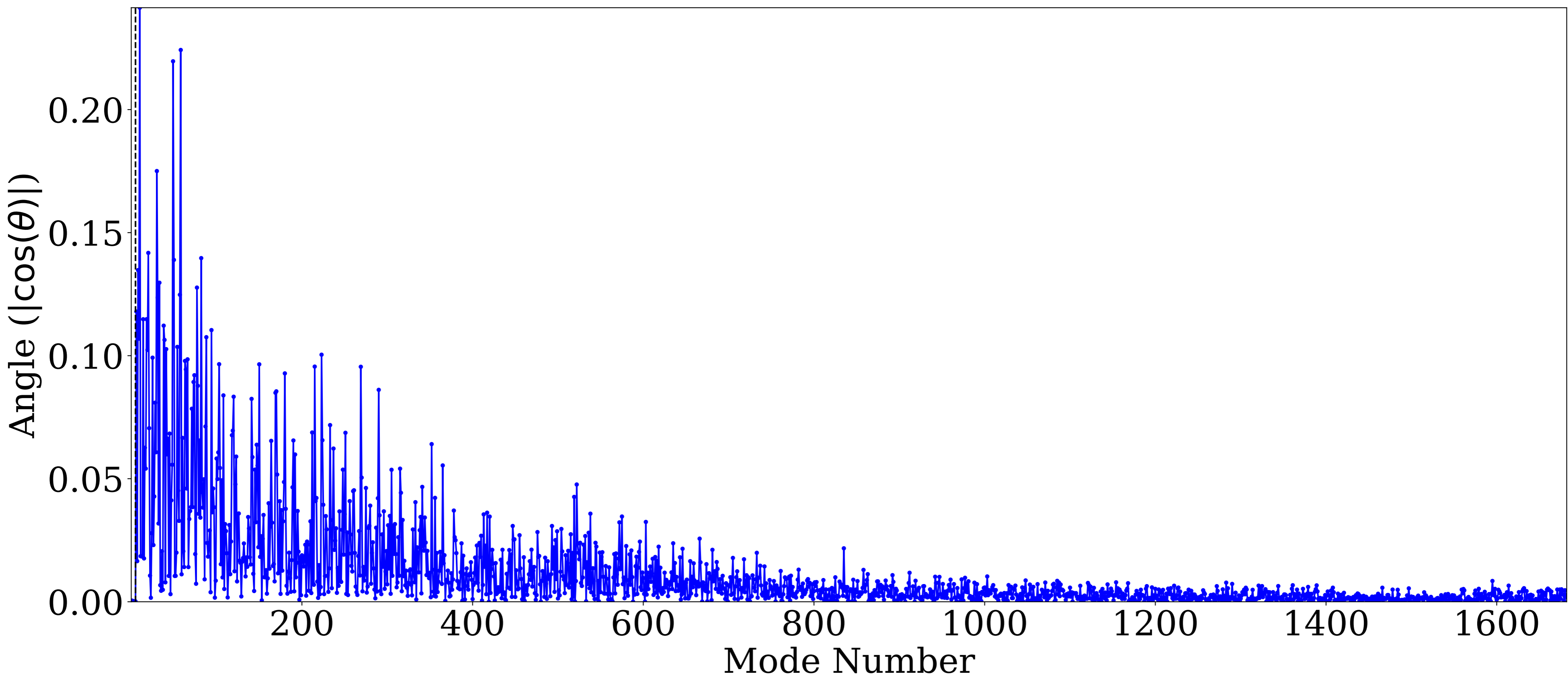

Supplement: NA-OLF-D6NA00012F-s001 [file NA-OLF-D6NA00012F-s001.zip › SupportingInformation/SI-Figures/min000000_3_561_DH.pdf]

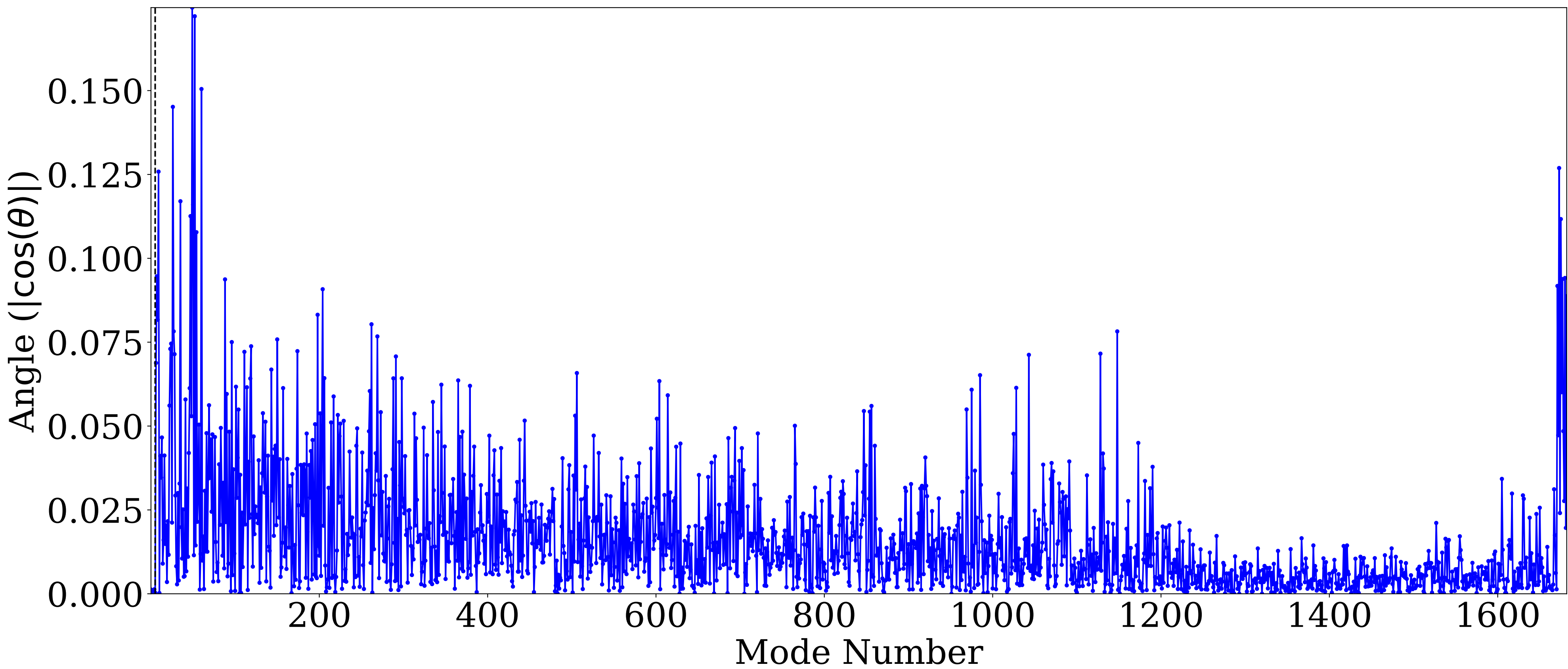

Supplement: NA-OLF-D6NA00012F-s001 [file NA-OLF-D6NA00012F-s001.zip › SupportingInformation/SI-Figures/min000000_3_561_FC.pdf]

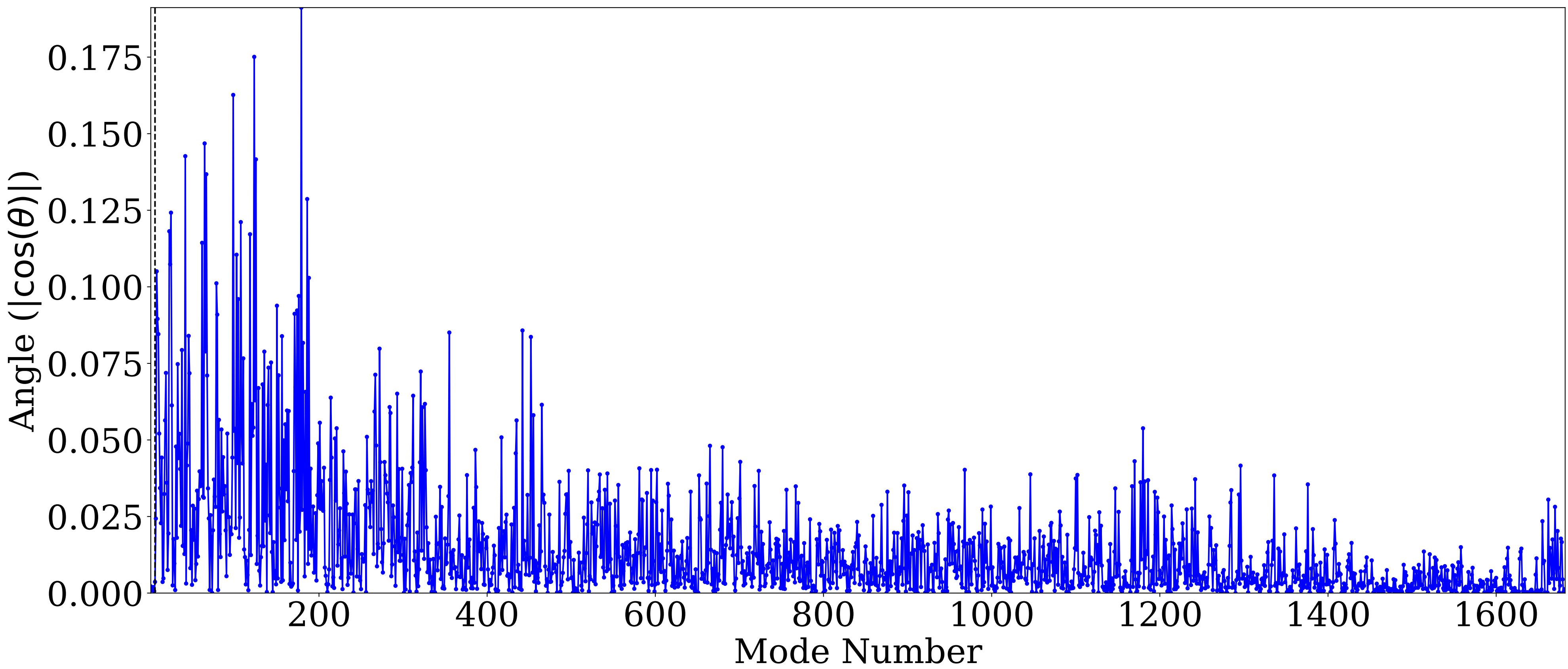

Supplement: NA-OLF-D6NA00012F-s001 [file NA-OLF-D6NA00012F-s001.zip › SupportingInformation/SI-Figures/min000000_3_561_IH.pdf]

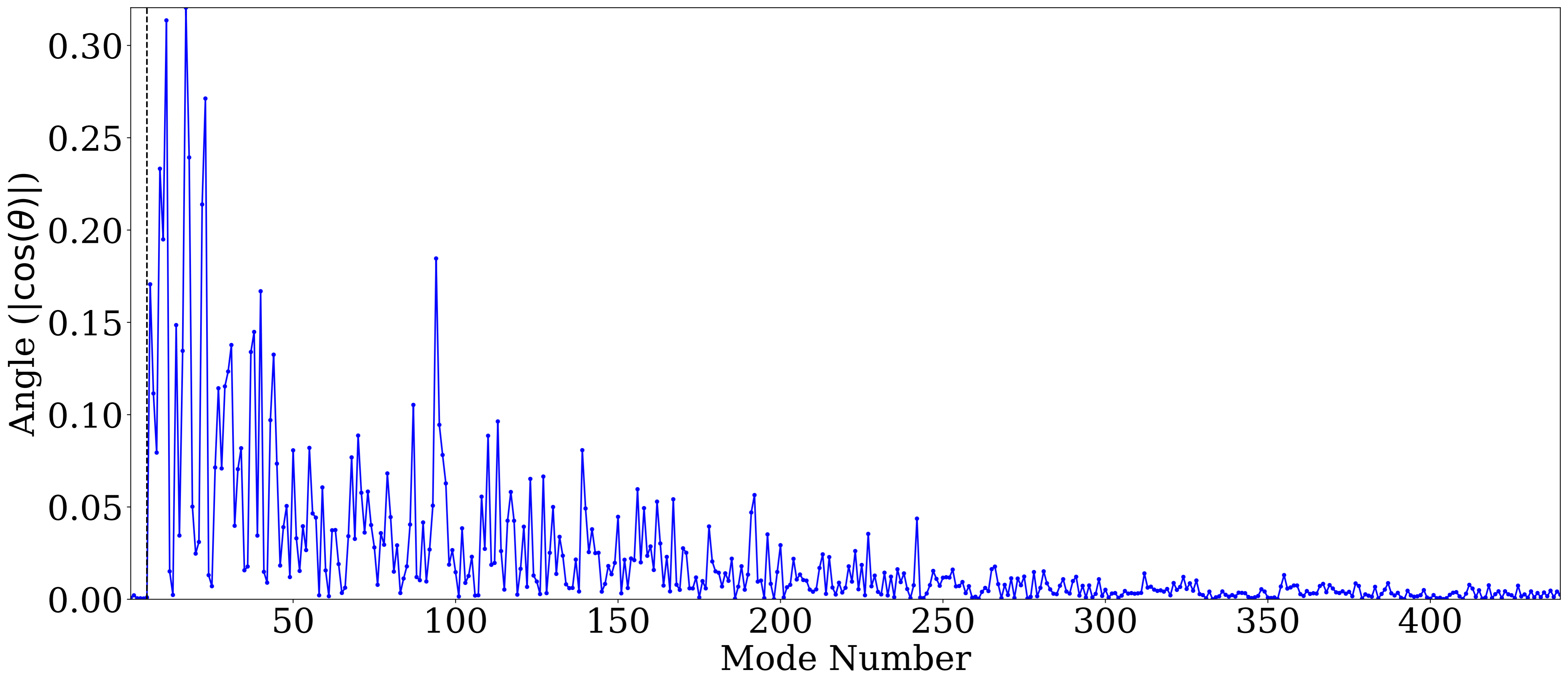

Supplement: NA-OLF-D6NA00012F-s001 [file NA-OLF-D6NA00012F-s001.zip › SupportingInformation/SI-Figures/min000003_1_147_DH.pdf]

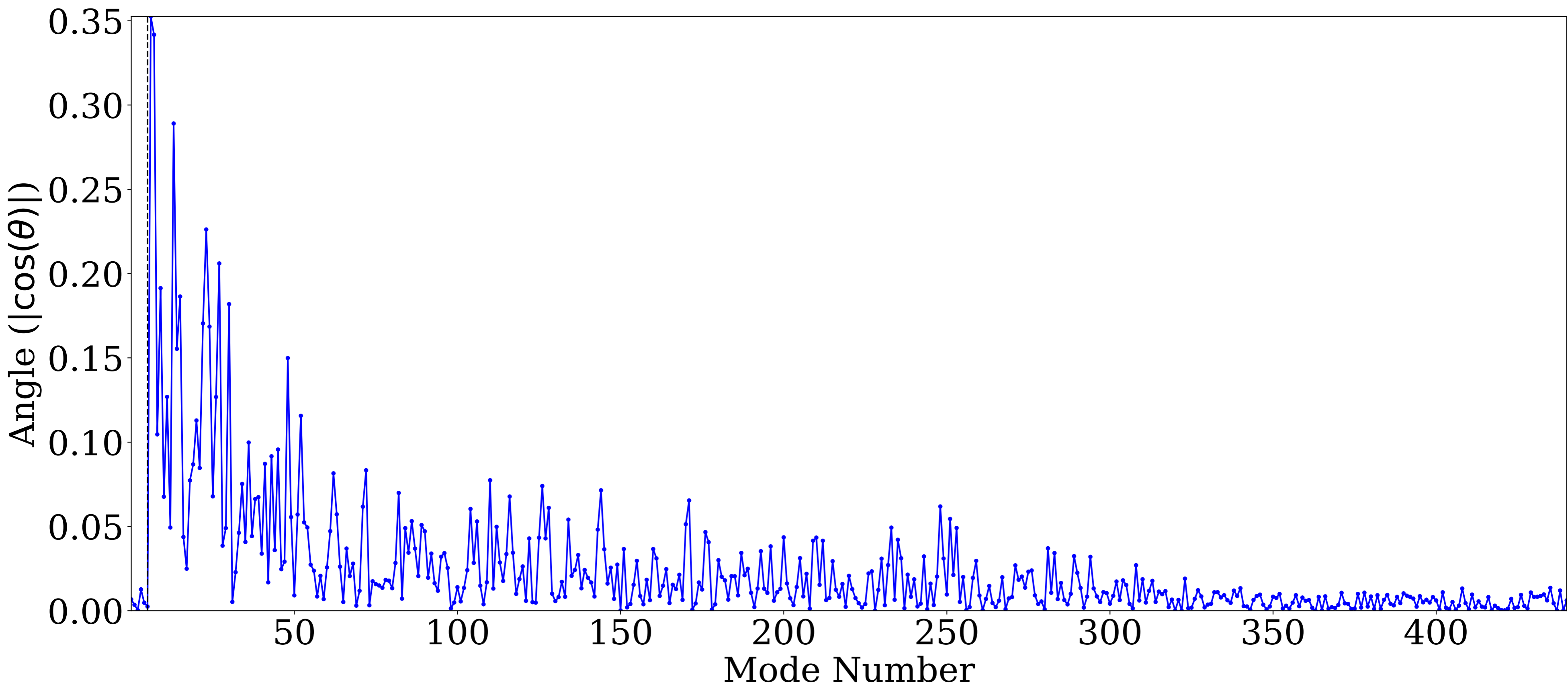

Supplement: NA-OLF-D6NA00012F-s001 [file NA-OLF-D6NA00012F-s001.zip › SupportingInformation/SI-Figures/min000003_1_147_FC.pdf]

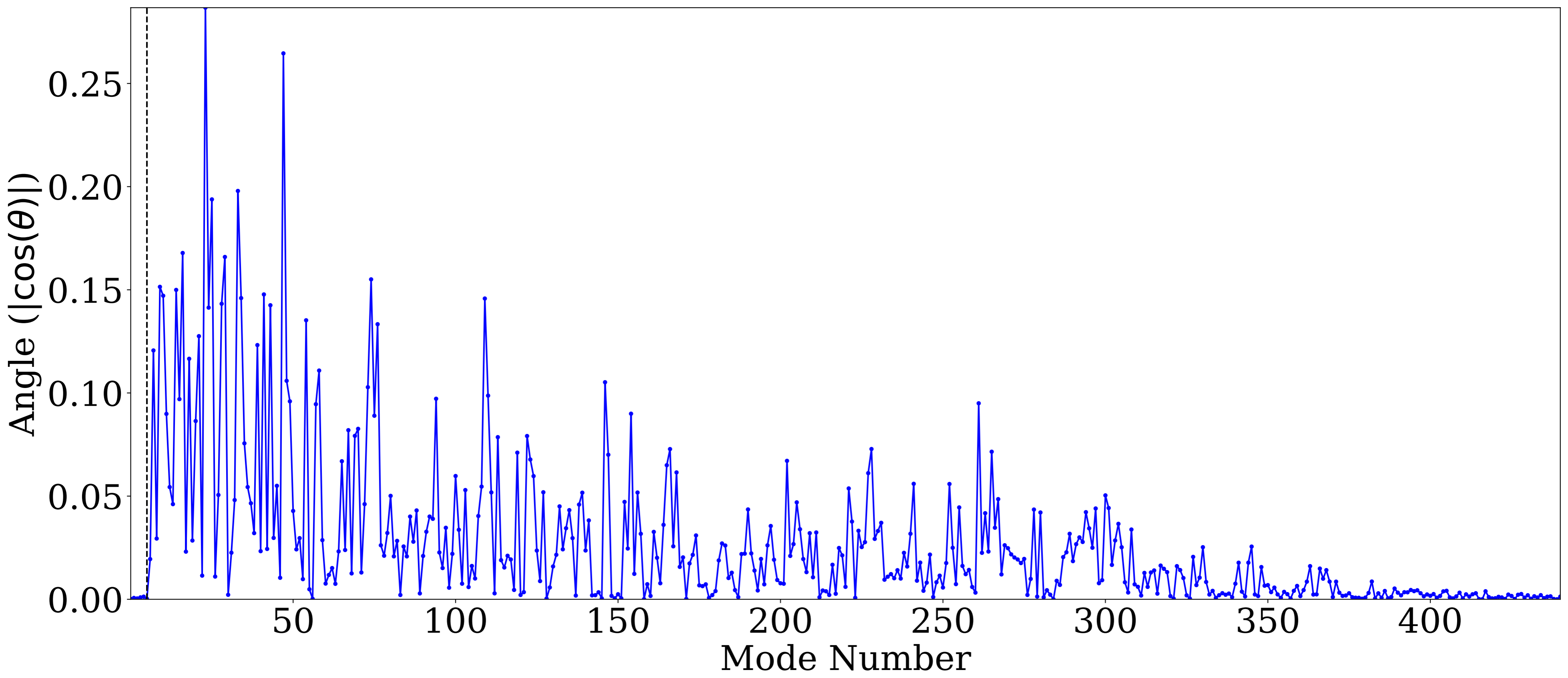

Supplement: NA-OLF-D6NA00012F-s001 [file NA-OLF-D6NA00012F-s001.zip › SupportingInformation/SI-Figures/min000003_1_147_IH.pdf]

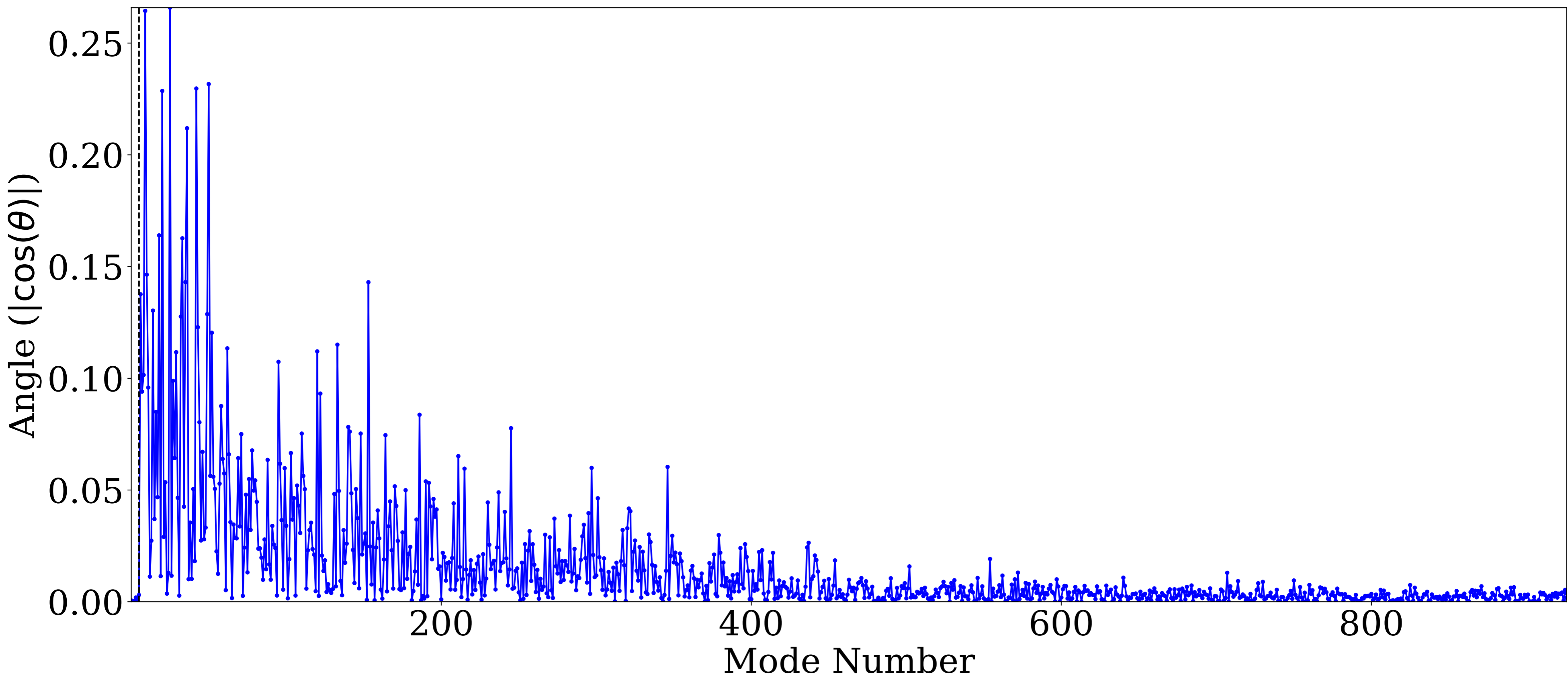

Supplement: NA-OLF-D6NA00012F-s001 [file NA-OLF-D6NA00012F-s001.zip › SupportingInformation/SI-Figures/min000005_1_309_DH.pdf]

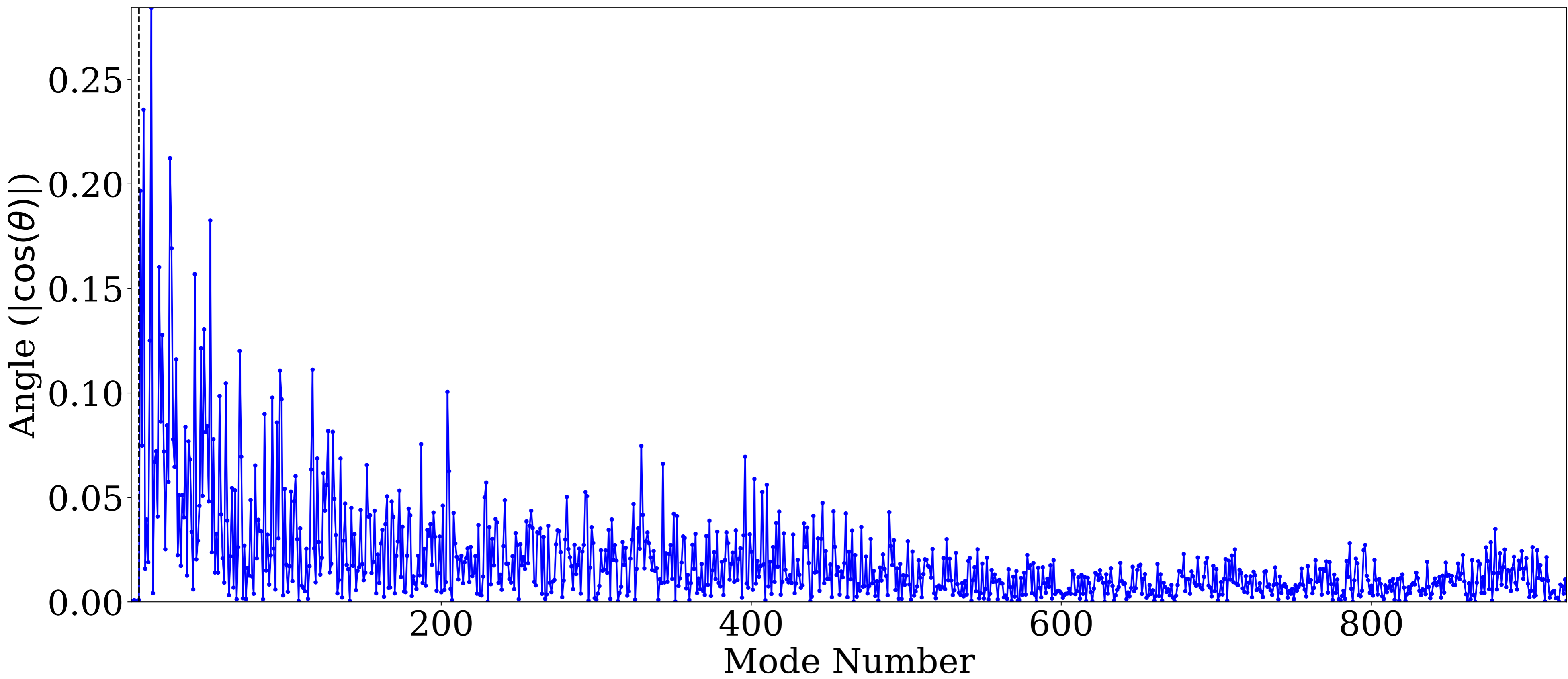

Supplement: NA-OLF-D6NA00012F-s001 [file NA-OLF-D6NA00012F-s001.zip › SupportingInformation/SI-Figures/min000005_1_309_FC.pdf]

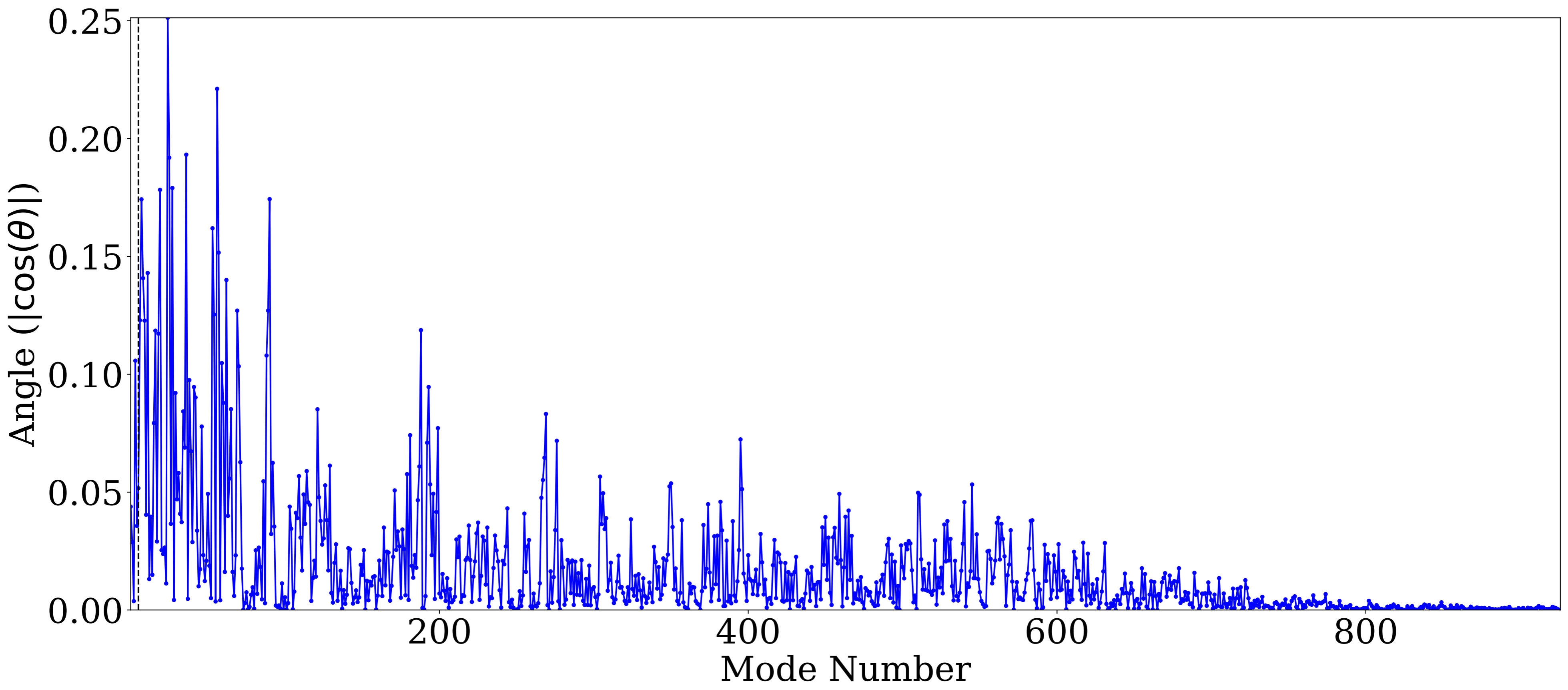

Supplement: NA-OLF-D6NA00012F-s001 [file NA-OLF-D6NA00012F-s001.zip › SupportingInformation/SI-Figures/min000005_1_309_IH.pdf]

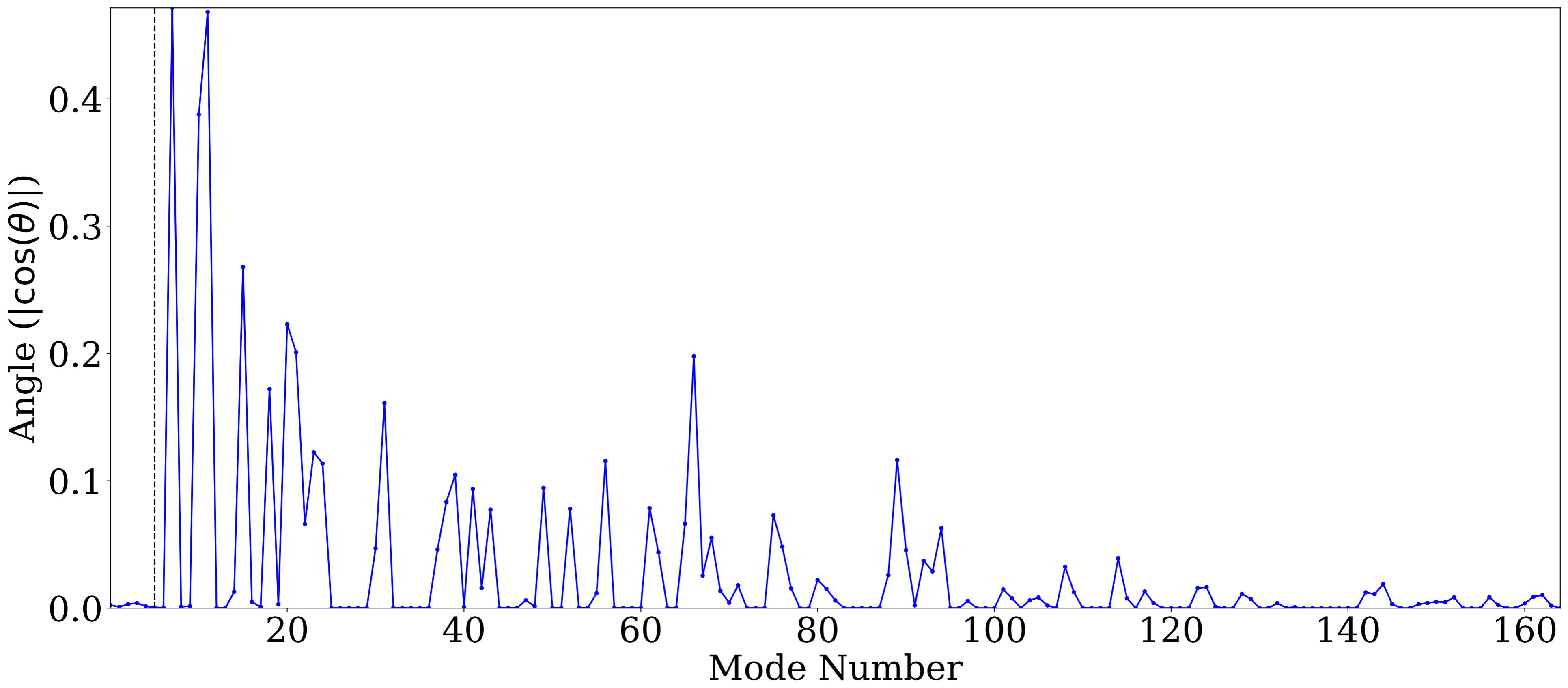

Supplement: NA-OLF-D6NA00012F-s001 [file NA-OLF-D6NA00012F-s001.zip › SupportingInformation/SI-Figures/min000009_1_055_DH.pdf]

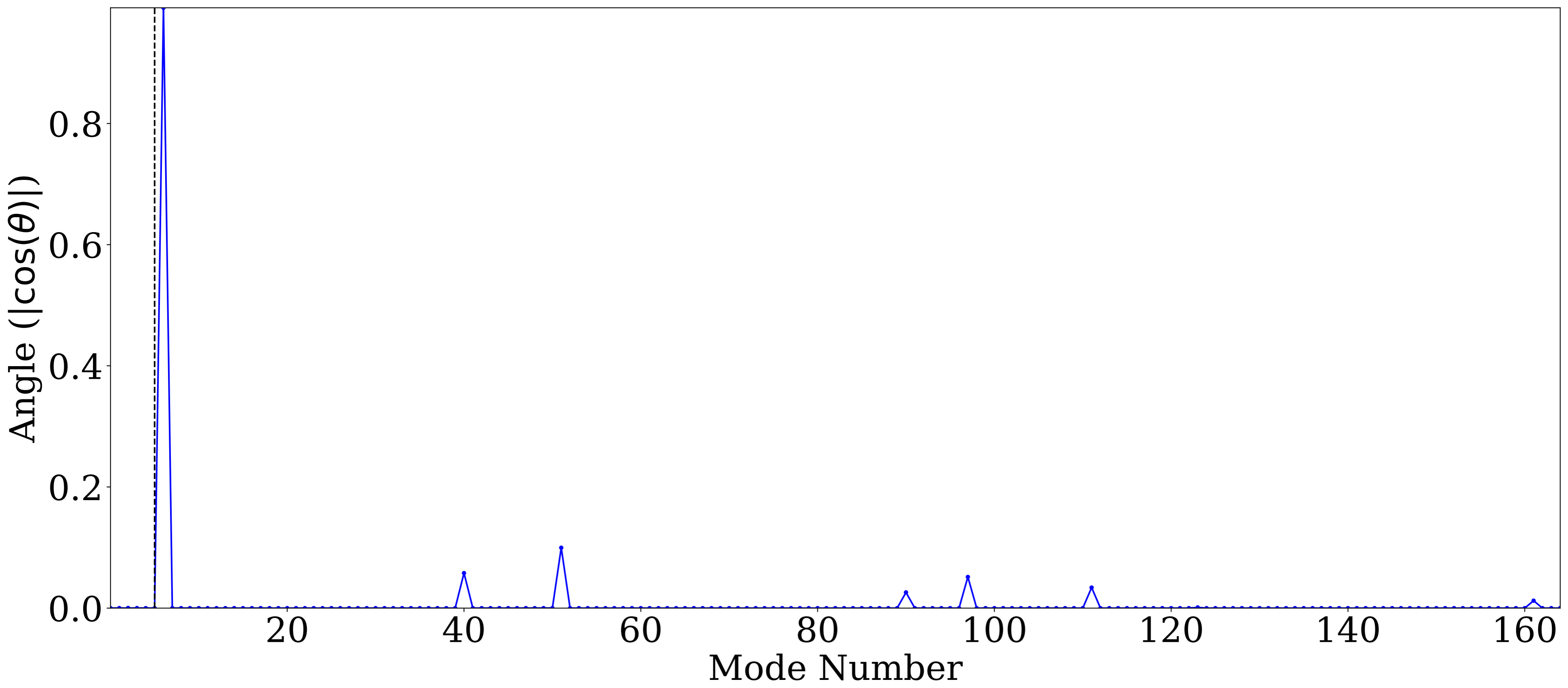

Supplement: NA-OLF-D6NA00012F-s001 [file NA-OLF-D6NA00012F-s001.zip › SupportingInformation/SI-Figures/min000009_1_055_FC.pdf]

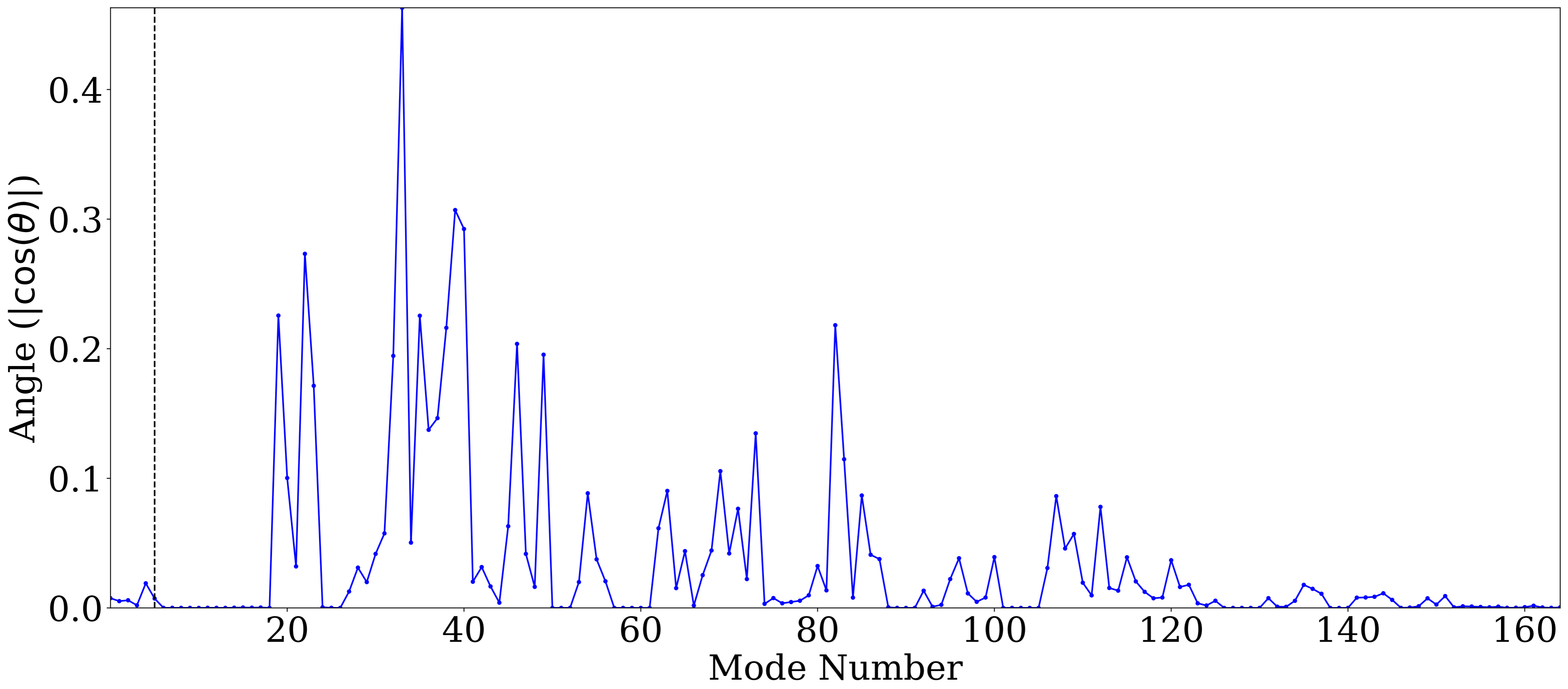

Supplement: NA-OLF-D6NA00012F-s001 [file NA-OLF-D6NA00012F-s001.zip › SupportingInformation/SI-Figures/min000009_1_055_IH.pdf]

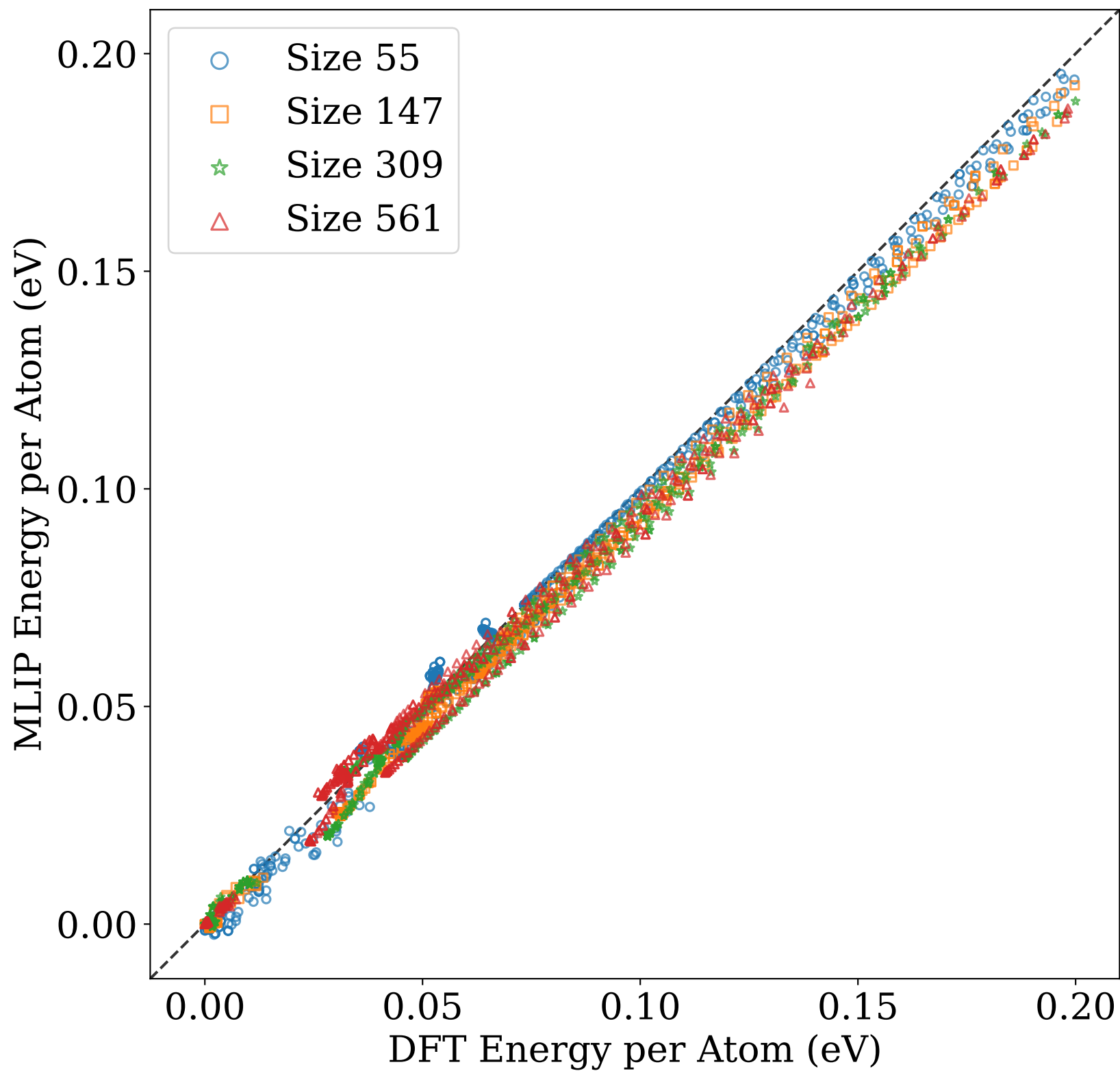

Supplement: NA-OLF-D6NA00012F-s001 [file NA-OLF-D6NA00012F-s001.zip › SupportingInformation/SI-Figures/total_ener_per_atom.pdf]
